# MiR-629-5p Promotes Prostate Cancer Development and Metastasis by Targeting AKAP13

**DOI:** 10.3389/fonc.2021.754353

**Published:** 2021-10-15

**Authors:** Yangzhou Liu, Shankun Zhao, Jiamin Wang, Zhiguo Zhu, Lianmin Luo, Qian Xiang, Mingda Zhou, Yuxiang Ma, Zuomin Wang, Zhigang Zhao

**Affiliations:** ^1^Department of Urology & Andrology, Minimally Invasive Surgery Center, Guangdong Provincial Key Laboratory of Urology, The First Affiliated Hospital of GuangZhou Medical University, Guangzhou, China; ^2^Department of Urology, Taizhou Central Hospital (Taizhou University Hospital), Taizhou, China; ^3^Department of Urology, Affiliated Hospital of Jining Medical University, Jining, China

**Keywords:** prostate cancer, miR-629-5p, AKAP13, phenotype, prognosis

## Abstract

Prostate cancer (PCa) has become the most frequently occurring cancer among western men according to the latest report, and patients’ prognosis is often poor in the event of tumor progression, therefore, many researches are devoted to exploring the molecular mechanism of PCa metastasis. MicroRNAs (miRNA) have proved to play an important role in this process. In present study, by combining clinical samples with public databases, we found that miR-629-5p increased to varying degrees in primary localized PCa tissues and metastatic PCa tissues compared with adjacent normal tissues, and bioinformatics analysis suggested that high level of miR-629-5p was related to poor prognosis. Functionally, miR-629-5p drove PCa cell proliferation, migration and invasion *in vitro*, and promoted growth of PCa cells *in vivo*. Moreover, A-kinase Anchor Protein 13 (AKAP13) was screened as a direct target of miR-629-5p, that expression was negatively correlated with the malignant phenotype of tumor cells. In the end, through verification in clinical specimens, we found that AKAP13 could be independently used as a clinical prognostic indicator. Overall, the present study indicates that miR-629-5p plays an oncogenic role in PCa by targeting AKAP13, which provides a new idea for clinical diagnosis and treatment of complex refractory PCa.

## Introduction

According to the latest research report, prostate cancer (PCa) has become the most frequently occurring cancer among western men, it accounts for about a quarter of all types of tumors (1). The incidence and mortality of PCa are also getting higher in China (2). Although clinical treatments such as surgery and endocrine therapy can effectively intervene the progression of PCa, patients’ prognosis is often poor in the event of tumor metastasis or castration resistance (3). Therefore, in the background of limited treatment, a better understanding of the molecular mechanism of PCa metastasis will contribute to the early diagnosis and intervention of patients with refractory PCa.

MicroRNAs (miRNAs) belong to endogenous small noncoding RNAs that post-transcriptionally regulate the gene expression by binding to the 3’-untranslated region (3’-UTR) of target mRNA, thereby inducing downstream mRNA degradation or protein synthesis repression (4). Accumulating researches have suggested that miRNAs play important roles in human cancer biological progression as novel types of tumor suppressors or oncogenes (5). A group of miRNAs, such as miR-146b, miR-210-3p, miR-636, miR-491-5p, have proved to play the driving role in the growth and metastasis of PCa (6–8). In addition, our previous studies have confirmed that miR-199b-5p and miR-671-5p can influence the metastasis of PCa by affecting epithelial-mesenchymal transition and transcriptional regulation, respectively (9, 10). Therefore, on this basis, we will further study the regulatory mechanism of miRNAs in the process of tumor metastasis.

By combining clinical samples with public databases, we found that miR-629-5p increased to varying degrees in primary localized PCa tissues (PPCa) and metastatic PCa tissues (MPCa) compared with adjacent normal tissues (ANT), and bioinformatics analysis suggested that high level of miR-629-5p was related to poor prognosis. Although miR-629-5p has proved to play a role in promoting tumor growth and metastasis in different types of tumors such as colorectal cancer, hepatocellular carcinoma, lung adenocarcinoma and osteosarcoma (11–14). However, the exact mechanism of its role in PCa metastasis remains unknown. In the present study, in addition to exploring the biological function of miR-629-5p as a key miRNA, we further confirmed that its mechanism is to promote the growth and metastasis of PCa by targeting inhibition of AKAP13, a tumor suppressor. These findings provide a clearer understanding of the mechanism of miRNA promoting PCa.

## Material and Methods

### Human Tissue Samples and Cell Lines

A total of 53 prostate clinicopathological samples were collected, including ANT (n = 13), PPCa tissue (n = 25) and MPCa tissue (n = 15), as reported in our previous research (10). This study was approved by the Ethics Committee of the First Affiliated Hospital for Guangzhou Medical University, and all patients have signed informed consents.

The normal human prostate epithelial cell line RWPE-1 and human PCa cell lines LNCaP, PC3, DU145 and VCaP were purchased from the Type Culture Collection of the Chinese Academy of Sciences (Shanghai, China). Human PCa cell line C4-2 and human embryonic kidney cell 293 T were obtained from the American Type Culture Collection (Manassas, VA, USA). All cell lines have been authenticated and were cultured as described previously (9).

### Public Database Analysis

The TCGA prostate adenocarcinoma (PRAD) database was acquired from the UCSC Xena database platform (http://xena.ucsc.edu/). The GSE21032 human PCa datasets were acquired from the GEO database (https://www.ncbi.nlm.nih.gov/geo/), which included two subsets: GSE21034 (mRNA sequencing data) and GSE21036 (miRNA sequencing data). The basic information of included datasets was presented in **Supplementary Table S1**. Overall, samples with complete clinical information and comprehensive matching gene expression were selected for subsequent analysis, and in order to ensure the validity of sufficient data, we chose biochemical recurrence (BCR, the amount of prostate-specific antigen in the blood has risen again after cancer treatment) as the endpoint event of prognosis index.

### RNA Transfection and Construction of Stable Cell Lines

In order to study the biological function of miRNA, we constructed a cell line stably transfected with lentivirus, including miR-629-5p overexpression cell line (named as miR-629-5p mimic: 5’-TGGGTTTACGTTGGGAGAACT-3’), miR-629-5p inhibition cell line (named as miR-629-5p inhibitor: 5’-AGTTCTCCCAACGTAAACCCA-3’) and the corresponding miRNA negative control (named as NC). Reagents used are all designed and constructed by GenePharma (Jiangsu, China). In addition, stable knockdown or knockout of AKAP13 expression PCa cells were established to further study the downstream target mRNA of miR-629-5p. The knockdown lentiviral vectors, which contain a specific shRNA (named as sh-AKAP13: target-1, 5’-GCAGCTCA ATTCCTAGCAACC-3’; target-2, 5’-GCTTCTAACCGAGGAGAATGC-3’; target-3, 5’-GCCAGTTCCCTGGATGGTAAC-3’; negative control named as sh-NC) against AKAP13 were synthesized by GenePharma (Jiangsu, China). The CRISPR/Cas9 knockout lentivirus (named as KO-AKAP13: target-1, 5’-CCAGAAGAGATGCTGCATCA-3’; target-2, 5’-ACTGGATCCGGTGATATCAC-3’; target-3, 5’-GTCCAGTGAAGCCGTGTCAT-3’; negative control named as KO-NC) was purchased from Guangzhou huiyuanyuan Co.ltd. (Guangzhou, China) (15), and the constructs were verified by DNA sequencing. Stable cell lines were selected for 10 days with Puromycin (PC-3, 2 μg/mL; LNCaP and C4-2, 4 μg/mL) or G418 (500 μg/mL). The transfection efficiency was identified by quantitative real-time polymerase chain reaction (qRT-PCR) and/or western blotting, and the most efficient virus was used in the following experiments.

### RNA Extraction and qRT-PCR

Total RNA was isolated using TRIzol (Invitrogen; Thermo Fisher Scientific, CA, USA). For miRNA quantification, TaqMan MicroRNA Reverse Transcription kit (Thermo Fisher Scientific Inc, MA, USA) and specific primers (RIBOBIO, Guangzhou, China) were used. For mRNA quantification, All-in-One First-Strand cDNA Synthesis kit (GeneCopoeia, Guangzhou, China) was used to prepare cDNA. qPCR for quantification of miRNA or gene expression was performed with SYBR green Premix Ex Taq II (Takara) on a CFX-96 system (Bio-Rad, Hercules, CA). The miRNA PCR thermal cycling conditions consisted of an initial denaturation at 95°C for 10 min, followed by 40 cycles of denaturation at 95°C for 10 s, annealing at 60°C for 20 s, and a 10 s extension at 72°C. The mRNA PCR protocol consisted of an initial denaturation at 95 °C for 15 min, followed by 40 cycles of denaturation at 95°C for 10 s, annealing at 58°C for 20 s, and a 30 s extension at 72°C. U6 and GAPDH were used as internal controls for miRNA and genes, respectively. Relative expression was determined by 2^-△△Ct^ method. Primer information was listed in **Supplementary Table S2**.

### Protein Extraction and Western Blotting

Total proteins were firstly extracted from cells, which were washed by cold PBS and treated with RIPA lysis buffer (KeyGEN, KGP703) (supplemented with protease inhibitors 1 mmol/L phenylmethylsulfonylfluoride, 10 mg/L pepstatin, 10 mg/L aprotinin and 5 mg/L leupeptin). Protein concentrations were determined using the BCA protein assay regents (#23225, Thermo Pierce, Rockford, IL, USA). The procedure of western blot was conducted as previously described (9). Primary antibodies used in the study include anti-AKAP13 (Immunoway, YT0161; Abcam, ab99377), anti-HEG1 (Biorbyt, orb157480), anti-caspase 3 (Immunoway, YT6113), anti-caspase 3 p17 (Immunoway, YT6161). Anti-β-tubulin (Abcam, ab210797) was used as internal standard. Detection was achieved in Odyssey CLX Two-color infrared laser imaging system (LI-COR Biosciences, Nebraska, USA). Densitometric analysis of the bands was performed using ImageJ software.

### Cell Proliferation Assays

The proliferation ability of PCa cells was measured by colony formation assay and 5‐ethynyl‐2′‐deoxyuridine (EdU) assay. For colony formation assay, 500 cells were seeded in 6-well plate and incubated in media containing 10% FBS for 2 weeks to allow colony formation. Then, colonies were fixed with 4% paraformaldehyde and stained with 0.1% crystal violet, and the results were recorded and counted (colony >50 cells). For EdU assay, cells were inoculated into a 24‐well plate, and the EdU kit (C10310-1, RIBOBIO, Guangzhou, China) was used to assess cell proliferations. The results were acquired using the fluorescent microscope (AX80, Olympus, Tokyo, Japan).

### Cell Migration and Invasion Assays

The migration and invasion ability of PCa cells were measured by wound-healing assays and transwell assay, respectively. The detailed procedure was conducted as described in our previous research (9, 16). For wound-healing assays, cells were plated into 6-well culture plates and cultured until 100% confluence, then the growth medium was removed, cells were washed and cultured with fresh serum-free medium, and the wound was produced by a 10μL sterile pipette tip. The transwell assay was using a transwell permeable support chamber (Corning Incorporated, Corning, NY, USA), which were coated with Matrigel (BD Biosciences), and was carried out according to the manufacturer’s instructions. Then, we observe cell migration/invasion and film the images under the optical microscope (CKX41, Olympus) at the indicated time points.

### Cell Apoptosis Assays

In order to study the effect of downstream gene expression changes on cell apoptosis, we first treated the KO-NC/KO-AKAP13 cell with 10µM Carbonyl cyanide 3-chlorophenylhydrazone (CCCP, solarbio) for 12 h to induce apoptosis, and then cells were harvested to detect apoptosis rate by Annexin V-FITC/propidium iodide (PI) Apoptosis Detection kit (Key Gen Biotech, Jiangsu, China). The experimental procedure was carried out according to the manufacturer’s protocol. Cytofluorimetric analysis was performed by the flow cytometer (Millipore, MA, USA).

### Target Gene Prediction and Dual-Luciferase Reporter Assays

Prediction of miR-629-5p target genes was accomplished by using miRWalk (http://mirwalk.umm.uni-heidelberg.de/), TarBase (http://carolina.imis.athena-innovation.gr/diana_tools/web/index.php?r=site%2Ftools), TargetScan (http://www.targetscan.org/vert_72/) and MicroT-CDS (http://diana.imis.athena-innovation.gr/DianaTools/index.php?r=microT_CDS/index) online tools. To further verify the target sites of miR-629-5p, dual-luciferase reporter assay was carried out. HK293T were plated in 96-well plates and transfected with pLUC-AKAP13-3’UTR-wild type (WT) or pLUC-AKAP13-3’UTR-mutant type (MUT) luciferase plasmids (GenePharma, Jiangsu, China) by lipofectamine 3000 (Invitrogen, Thermo Fisher Scientific, Inc.). After 48 h, Luciferase and Renilla signals were measured using Dual-Luciferase Reporter Assay System (Promega, Madison, WI, USA).

### Animal Experiments

Thirty 5 weeks old male BALB/c nude mice were purchased from the Experimental Animal Center of Guangdong Province (Guangzhou, China). The animals were fed as described previously (9). All procedures related to the experimental animals were approved by the Animal Care and Use Committee of the First Affiliated Hospital of Guangzhou Medical University.

To evaluate the effects of miR-629-5p on tumor growth, mice were randomly divided into six groups (NC/mimic/inhibitor group, n=5/group) and each mouse was subcutaneously injected with concentrated tumor cells 2×10^6^ to establish xenograft tumors. The tumor sizes were monitored weekly. After 4 weeks, the mice were sacrificed by cervical dislocation, and the tumors were dissected and weighed. The tissue was fixed and embedded in paraffin wax for histological examination and immunohistochemical (IHC) assay.

### Histological and Immunohistochemical Assessment

Histological and IHC analysis were performed in mice xenografts and clinical PCa samples. The tissue morphology was observed by haematoxylin and eosin (H&E) staining, and the expression of target protein in different tissues was evaluated by IHC. The procedure of H&E and IHC were carried out as described previously (17). For IHC, primary antibodies used in the study include anti-AKAP13 (Novus, NBP1-89163) and anti-KI67 (Servicebio, GB111499). When the experiment was complete, the results were observed and recorded under the optical microscope (CKX41, Olympus), and the expression intensity of target protein was quantified according to the previous protocol (17).

### Statistical Analyses

The SPSS V24.0 software (SPSS Inc., Chicago, IL, USA) and GraphPad Prism 7.0 (San Diego, CA, USA) were used to perform statistical analyses. For continuous variables, data were presented as the means ± SD. Comparison between groups was carried out using the Student’s t test, paired t-test or one way ANOVA. The Fisher’s exact test was used for 2 × 2 tables. Spearman’s correlations were calculated for the expression levels between miR-629-5p and target gene. Survival curves were plotted using Kaplan-Meier’s method and compared between groups by the log-rank test. X-tile program was used to determine the cut-off values which optimized the significance of the split between Kaplan-Meier survival curves (18). P < 0.05 was considered statistically significant.

## Results

### Identify and Verify miRNA Associated With PCa Progression by Bioinformatics Analysis

**Figure 1:**
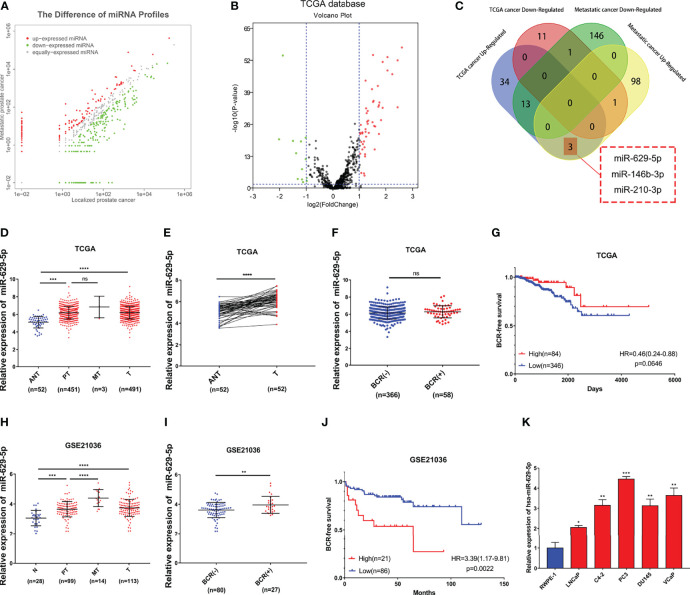
Identify and verify miRNA associated with PCa progression by bioinformatics analysis. **(A)** The differential expression miRNA of clinical samples (102 upregulated/160 downregulated miRNAs related to PCa metastasis, threshold set as |log2(Fold Change)|≥1, P < 0.05). **(B)** The differential expression miRNA of TCGA-PRAD database (50 upregulated/13 downregulated miRNAs related to PCa formation, threshold set as |log2(Fold Change)|≥1, P < 0.05). **(C)** Identify key miRNAs related to PCa progression by combining clinical samples with TCGA database. **(D)** miR-629-5p expression increased in primary localized PCa tissues and metastatic PCa tissues compared with adjacent normal tissues in TCGA. **(E)** miR-629-5p expression levels was upregulated in 52 paired PCa tissues compared with that in the matching adjacent normal tissues in TCGA. **(F)** Correlations between miR-629-5p expression and patients’ BCR status in TCGA. **(G)** The Kaplan-Meier survival analysis of BCR-free survival about miR-629-5p expression in TCGA. **(H)** miR-629-5p expression increased in primary localized PCa tissues and metastatic PCa tissues compared with normal tissues in GSE21036. **(I)** Correlations between miR-629-5p expression and patients’ BCR status in GSE21036. **(J)** The Kaplan-Meier survival analysis of BCR-free survival about miR-629-5p expression in GSE21036. **(K)** Real-time PCR analysis of miR-629-5p expression in normal prostate epithelial cell (RWPE-1) and PCa cells. The data were presented as means ± SD from three biological replicates. ^ns^P > 0.05; *P < 0.05; **P < 0.01; ***P < 0.001; ****P < 0.0001; Student’s t-test **(D, F, H, I, K)**; paired t-test **(E)**. ANT, adjacent normal tissues; N, normal tissues; PT, primary localized PCa tissues; MT, metastatic PCa tissues; T, tumor tissues; BCR, biochemical recurrence; HR, hazard ratio.

By combining the miRNA sequencing result of clinical samples in our previous research (9) (**Figure 1A** and **Supplementary Table S3**, 102 upregulated differential expression miRNA/160 downregulated differential expression miRNA related to PCa metastasis) with it in the TCGA-PRAD database (**Figure 1B**, **Supplementary Table S4**, 50 upregulated differential expression miRNA/13 downregulated differential expression miRNA related to PCa formation), we have identified four key differential expression miRNA related to the progression of PCa (**Figure 1C**, 3 upregulated: miR-629-5p, miR-146b-3p, miR-210-3p; 1 downregulated: miR-221-3p). By consulting relevant literature, we found that the mechanism of miR-146b-3p, miR-210-3p and miR-221-3p in PCa has been reported (6, 7, 19), which further confirmed the reliability of our screening results from the side. Therefore, we chose miR-629-5p for verification in the follow-up study.

First, we found that miR-629-5p expression increased to varying degrees in primary localized PCa tissues and metastatic PCa tissues compared with adjacent normal tissues, and it had a consistent trend between TCGA tumor and adjacent normal tissues paired samples (**Figures 1D, E**). The same result was also verified in GSE21036 dataset (**Figure 1H**). At the same time, we found that miR-629-5p expression was upregulated in most other tumors by using dbDEMC 3.0 (**Supplementary Table S5**) (20). Subsequently, in order to test whether miR-629-5p can be used as a potential clinical prognostic indicator, we explored the association between miR-629-5p expression and patients’ BCR and whether it has survival prognostic value. The results in GSE21036 dataset show that the expression of miR-629-5p and patients’ BCR were related, and high miR-629-5p expression associated with shorter BCR-free survival, which suggested that high expression of miR-629-5p predicted poor prognosis (**Figures 1I, J**). However, the same result was not found in TCGA-PRAD database (**Figures 1F, G**). Finally, we examined miR-629-5p expression in prostate related cell line, and found it was significantly high expression in PCa cells compared with normal prostate epithelial cell line RWPE-1, especially in higher malignant degree PC3 cell lines. (**Figure 1K**).

### MiR-629-5p Facilitates PCa Cells Proliferation, Migration, and Invasion *In Vitro*

**Figure 2:**
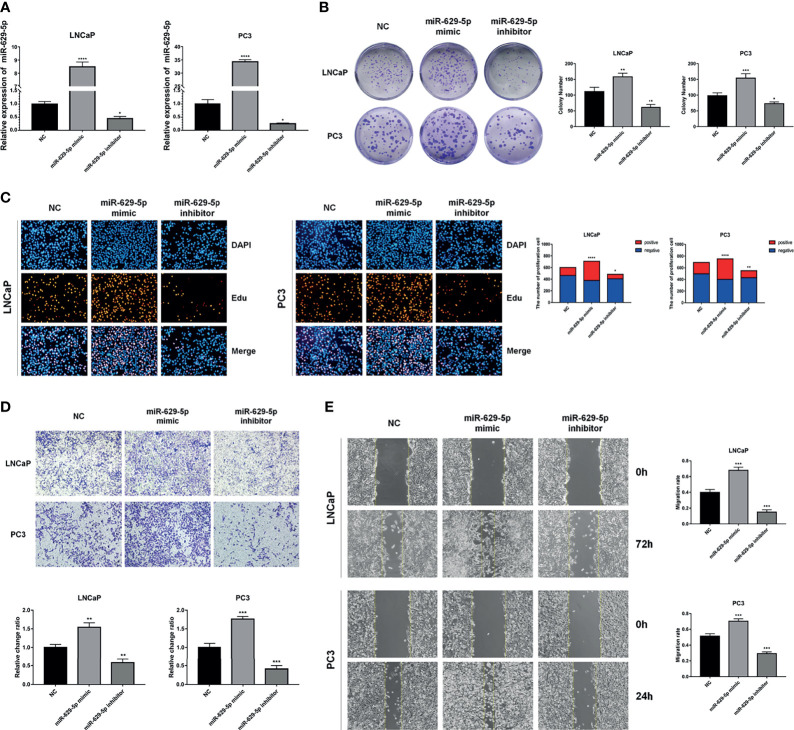
MiR-629-5p facilitates PCa cells proliferation, migration, and invasion *in vitro*. **(A)** Stable miR-629-5p overexpression or inhibition PCa cell lines were established and confirmed by qRT-PCR. **(B)** Colony formation assays and **(C)** EdU assays were performed to assess the proliferation ability changes in miR-629-5p overexpressing or inhibitory cell lines. **(D)** Transwell assays and **(E)** Wound-healing assays were performed to assess the invasion and migration ability changes in miR-629-5p overexpressing or inhibitory cell lines. The data were presented as means ± SD from three biological replicates. *P < 0.05; **P < 0.01; ***P < 0.001; ****P < 0.0001; Student’s t-test.

In order to investigate the biological functions of miR-629-5p in PCa, we selected PCa cell lines with the lowest (LNCaP) and highest (PC3) miRNA expression levels as the research objects, and manipulated the expression level of miRNA by stably transfecting lentivirus. First, qPCR was conducted to confirm the transfection efficiency (**Figure 2A**). Colony formation and EdU assays were performed to assess the influence of miR-629-5p on the proliferation ability of PCa cells. The Colony formation assay revealed that the cell viability was prominently enhanced by miR-629-5p mimics, while miR-629-5p inhibitors inhibited the viability (**Figure 2B**). The corresponding change in the percentage of positive cells in EDU assay also supports this result (**Figure 2C**). Then, the migration and invasion ability of PCa cells were measured by wound-healing assays and transwell assays, respectively. To put it simply, over-expressing miR-629-5p dramatically facilitated the migratory and invasive capacities of cell, while inhibit miR-629-5p significantly suppressed these capacities (**Figures 2D, E**). These results suggested that miR-629-5p acts as an oncogenic miRNA in PCa cells *in vitro*.

### MiR-629-5p Promotes Tumor Growth *In Vivo*

**Figure 3:**
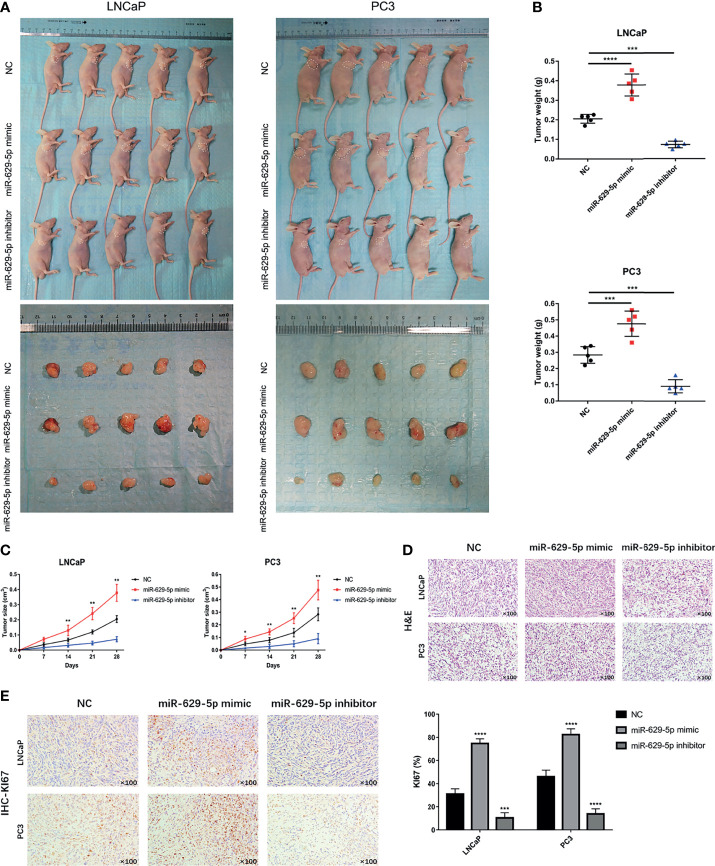
MiR-629-5p promotes tumor growth *in vivo*. **(A)** The subcutaneous xenograft tumor model in nude mice was established, and the representative appearance of tumor mass resected from each group of mice. **(B)** Final tumor weights were measured at autopsy on day 28 after subcutaneous injection stable transfected PCa cells. **(C)** The tumor growth curves were measured with a calliper at the indicated days after cell injecting. **(D)** The xenograft tumor tissues were stained with H&E. **(E)** KI67 IHC staining was performed in xenograft tumor tissues to assess tumor proliferation. Magnification, ×100. The data were presented as means ± SD from three biological replicates. *P < 0.05; **P < 0.01; ***P < 0.001; ****P < 0.0001; Student’s t-test.

To investigate the effect of miR-629-5p on PCa cell proliferation *in vivo*, we established a subcutaneous xenograft tumor model in nude mice (**Figure 3A**). The results show that, compared with NC group, the cell lines overexpressing miRNA had faster tumor growth rate and larger weight, while the cell lines silencing miRNA showed the opposite trend (**Figures 3B, C**). H&E staining showed the histopathological features of the tumor tissues (**Figure 3D**). Furthermore, we further verified the change of tumor growth ability by cell proliferation marker KI67 immunostaining (**Figure 3E**). Taken together, *in vivo* study also confirmed the correlation between miR-629-5p and tumor malignancy.

### AKAP13 Is a Direct Function Target of miR-629-5p

**Figure 4:**
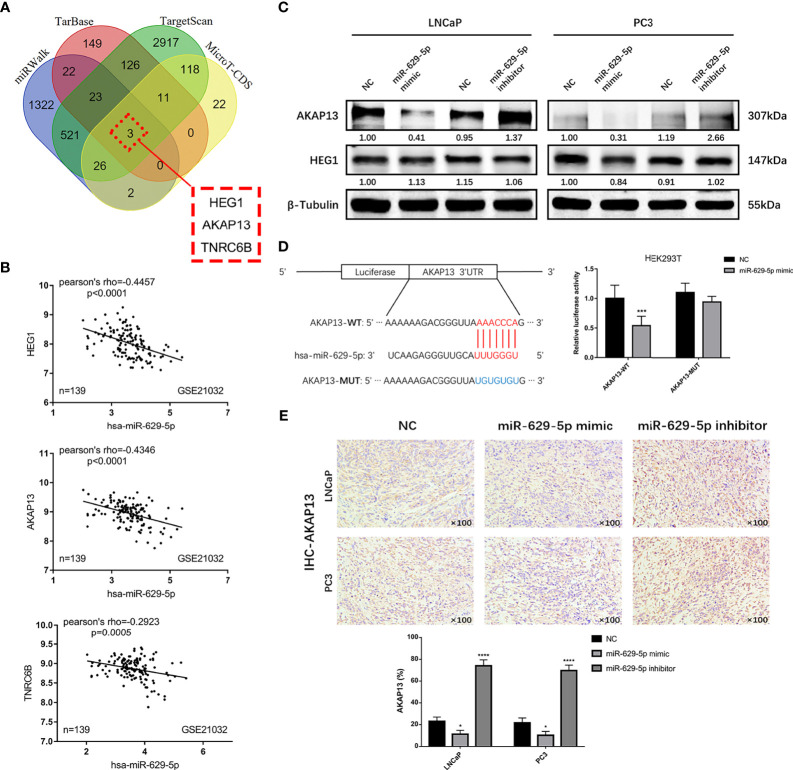
AKAP13 is a direct function target of miR-629-5p. **(A)** Identify potential miR-629-5p target genes by four common miRNA prediction tools. **(B)** The correlations between miR-629-5p expression and HEG1, AKAP13 and TNRC6B expression in GSE21032. **(C)** Western blotting was carried out to verify the expression of HEG1 and AKAP13 at protein level in stable transfected PCa cells, merely AKAP13 could be significantly regulated by miR-629-5p, but not HEG1. **(D)** Left: potential miR-629-5p binding sites in the 3’UTR of AKAP13 mRNAs. Right: the dual-luciferase reporter assays showed that miR-629-5p overexpression significantly reduced the luciferase activity of binding site of AKAP13, and the mutation of binding site blocked the interaction. **(E)** AKAP13 IHC staining was performed in xenograft tumor tissues. Magnification, ×100. The data were presented as means ± SD from three biological replicates. *P < 0.05; ***P < 0.001; ****P < 0.0001; Student’s t-test. WT, wide type; MUT, mutant type.

MiRNA has proved to play a biological function by regulating the expression of target genes. In order to clarify its mechanism, we screened out three candidate genes (HEG1, AKAP13 and TNRC6B) through the tool of target gene prediction (**Figure 4A**). Among them, HEG1 and AKAP13 were found in GSE21032 database and have better correlation with miR-629-5p (Pearson’s rho = -0.4457 and -0.4346, **Figure 4B**). However, merely AKAP13 could be significantly regulated by miR-629-5p at the protein level (**Figure 4C**). To further verify the regulatory relationship, we analyzed the existence of miR-629-5p binding sites in the 3’UTR region of AKAP13, and based on this, we designed dual-luciferase reporter assays. The overexpression of miR-629-5p significantly reduced the luciferase activity of binding site of AKAP13, and the mutation of binding site blocked the interaction (**Figure 4D**). Which proved that AKAP13 is a direct target of miR-629-5p. IHC staining of xenografts also revealed that AKAP13 expression was regulated by miR-629-5p (**Figure 4E**).

### AKAP13 Is Essential for miR-629-5p Enhanced Cell Proliferation and Motility in PCa

**Figure 5:**
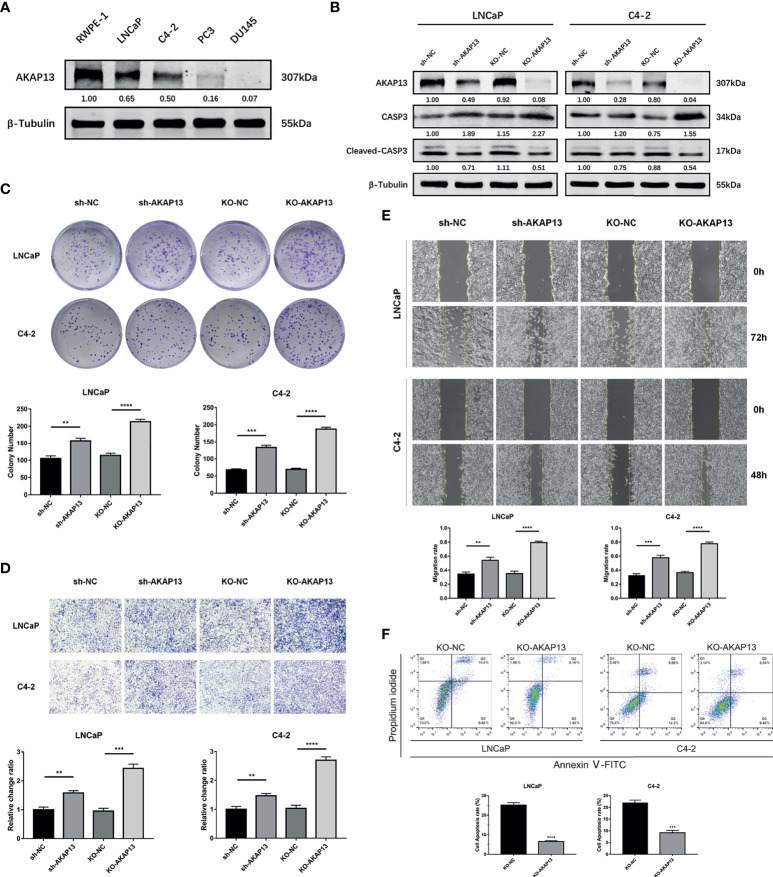
AKAP13 expression changes can affect the malignant phenotype of PCa cells. **(A)** Western blotting analysis of AKAP13 protein expression in RWPE-1 and PCa cells. **(B)** Stable AKAP13 knockdown or knockout PCa cell lines were established, and the sequence with the highest inhibitory efficiency was verified by Western blotting. After adding CCCP to stably transfected cell lines to induce apoptosis, the expression of apoptosis-related proteins changed. **(C)** Colony formation assays were performed to assess the proliferation ability changes in AKAP13 inhibitory cell lines. **(D)** Transwell assays and **(E)** Wound-healing assays were performed to assess the invasion and migration ability changes in AKAP13 inhibitory cell lines. **(F)** After adding CCCP to stably AKAP13 knockout cell lines to induce apoptosis, then cell apoptosis rate was determined by Annexin V-FITC/PI staining. The data were presented as means ± SD from three biological replicates. **P < 0.01; ***P < 0.001; ****P < 0.0001; Student’s t-test. CCCP, Carbonyl cyanide 3-chlorophenylhydrazone.

Through the detection of AKAP13 protein in prostate related cell lines, high-level expression in RWPE-1 indicates that it might be a potential tumor suppressor gene (**Figure 5A**). In order to validate whether AKAP13 is the downstream effector of miR-629-5p in PCa, the expression was downregulated by transfecting knockdown/knockout lentivirus (**Figure 5B**). Particularly, in order to make the effect of gene down-regulation more obvious, we selected LNCaP and C4-2 cell line as the follow-up research objects. Subsequently, the increase of cell proliferation and metastasis ability in different degrees demonstrated the negative regulation effect of AKAP13 on cell malignant phenotype (**Figures 5C–E**). In addition, compared with the control group, gene knockout can increase the tolerance of cells to apoptosis induction (**Figure 5F**). The corresponding changes of apoptosis-related proteins expression also verified this finding (**Figure 5B**).

**Figure 6:**
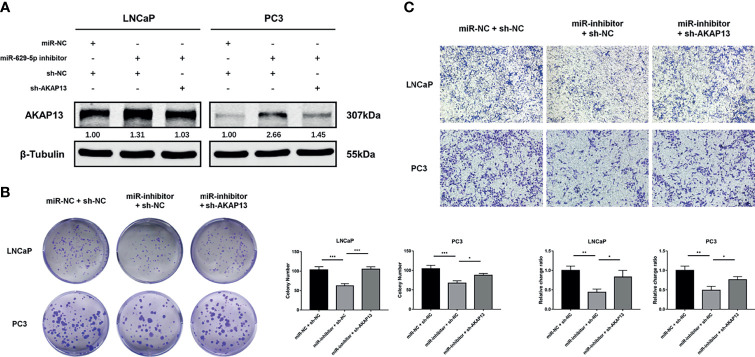
AKAP13 knockdown rescues the miR-629-5p inhibition-attenuated PCa cell proliferation and motility. **(A)** AKAP13 was knocked down by the shRNA in PCa cells with miR-629-5p inhibition, and the transfection efficiency was confirmed by western blotting. **(B)** Colony formation assays and **(C)** Transwell assays were performed to assess the proliferation and invasion abilities of PCa cells transfected with the corresponding vectors. The data were presented as means ± SD from three biological replicates. *P < 0.05; **P < 0.01; ***P < 0.001. Student’s t-test.

To further confirm whether miR-629-5p promoted PCa development through AKAP13, we performed rescue experiment of AKAP13 knockdown in corresponding cells with stable miRNA inhibited (**Figure 6A**). As shown in **Figures 6B, C**, after gene double knockdown, the proliferation and metastasis ability of PCa cells partially recovered, the anticancer effect of miR-629-5p inhibitor could be reversed. Our results suggested that AKAP13 is essential for miR-629-5p takes effect in PCa progress.

### AKAP13 Expression Is Negatively Correlated With PCa Malignant Degree and Correlate With Clinical Outcomes of PCa Patients

**Figure 7:**
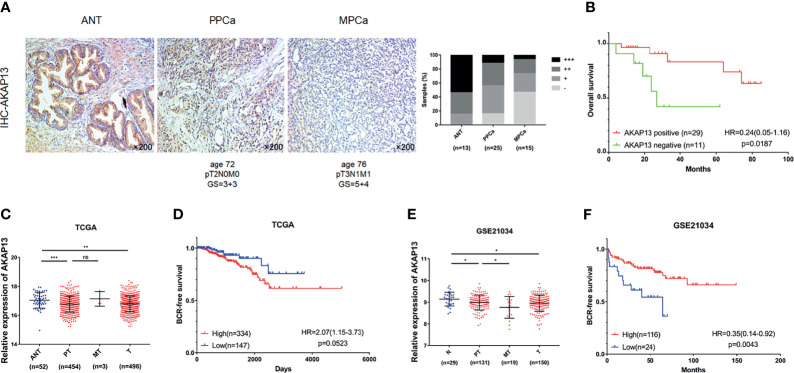
AKAP13 expression is negatively correlated with PCa malignant degree and correlate with clinical outcomes of PCa patients. **(A)** AKAP13 IHC staining was performed in clinical adjacent normal tissues (n = 13) and PCa tissue samples (n = 40). Left: representative IHC photographs of ANT and tumor tissue samples (PPCa, MPCa) were shown as indicated. Right: the composition of AKAP13 different expression degree (-: negative expression; +: weak expression; ++: moderate expression; +++: strong expression) in each group of tissue samples was counted. **(B)** The Kaplan-Meier survival analysis of overall survival of PCa patients with different AKAP13 expression levels. **(C)** AKAP13 expression decreased in PCa tumor tissues compared with adjacent normal tissues in TCGA. **(D)** The Kaplan-Meier survival analysis of BCR-free survival about AKAP13 expression in TCGA. **(E)** AKAP13 expression decreased in primary localized PCa tissues and metastatic PCa tissues compared with normal tissues in GSE21034. **(F)** The Kaplan-Meier survival analysis of BCR-free survival about AKAP13 expression in GSE21034. Magnification, ×200. The data were presented as means ± SD. ^ns^P > 0.05; *P < 0.05; **P < 0.01; ***P < 0.001; Student’s t-test. ANT, adjacent normal tissues; N, normal tissues; PPCa/PT, primary localized PCa tissues; MPCa/MT, metastatic PCa tissues; T, tumor tissues; BCR, biochemical recurrence; HR, hazard ratio.

Immunohistochemical staining was performed on enrolled 53 clinical samples. By evaluating and counting the staining results, we get the number of AKAP13 different expression (-: negative expression; +: weak expression; ++: moderate expression; +++: strong expression) in three groups of patients (ANT, PPCa and MPCa) (**Figure 7A**). As shown in **Table 1**, combined with the clinicopathological characteristics of patients, we found that the expression level of AKAP13 has a significant correlation with the patients’ part clinical indicators, such as gleason score (P=0.006) and distant metastasis (P=0.029). At the same time, by analyzing the survival data of patients, we found that AKAP13 negative patients had shorter overall survival time (**Figure 7B**), which suggested that AKAP13 expression is negatively correlated with PCa malignant degree and could be used as a potential clinical prognostic indicator. Finally, we verify this result by analyzing the public database (TCGA-PRAD database and GSE21034 dataset) (**Figures 7C–F**).

**Table 1:** Correlation between AKAP13 expression with clinicopathological characteristics in PCa.

Variables	n	AKAP13		p^a^
		Positive (%)	Negative (%)	
Patients	40	29 (72.5)	11 (27.5)	
Age (year)				
≤72	19	15 (78.9)	4 (21.1)	0.488
>72	21	14 (66.7)	7 (33.3)	
GS				
<7	22	20 (90.9)	2 (9.1)	0.006
≥7	18	9 (50.0)	9 (50.0)	
pT				
≤T2	20	12 (60.0)	8 (40.0)	0.155
T3–T4	20	17 (85.0)	3 (15.0)	
pN				
N0	28	21 (75.0)	7 (25.0)	0.704
N1	12	8 (66.7)	4 (33.3)	
M				
M0	26	22 (84.6)	4 (15.4)	0.029
M1	14	7 (50.0)	7 (50.0)	

AKAP13 expression was determined by IHC.

GS, gleason score; pT, pathologic tumor stage; pN, pathologic lymph node metastasis; M, distant metastasis.

a Chi-square test.

## Discussion

Because of the complexity of mechanism, tumor metastasis often means higher medical expenses and worse quality of life for patients. Therefore, many researches are devoted to exploring the molecular mechanism of PCa metastasis. More and more miRNAs have been shown to participate in various tumor processes, including tumor initiation, progression, and metastasis. In this research, by combining clinical samples with public databases, we found a novel miRNA (miR-629-5p) related to the occurrence and development of PCa, and proved that its expression is closely associated with the malignant phenotype of tumor on cell and animal models. On this basis, we searched for the downstream target gene (AKAP13) of miR-629-5p, and confirmed that it played biological function through this key gene. Finally, the potential value of AKAP13 as a tumor suppressor gene was verified in clinical data.

The miR-629-5p has been reported to play an oncogenic in many types of cancer. In renal cell carcinoma, Kentaro et al. have proved that miR-629 could promote TGFβ/Smad Signaling and tumor metastatic phenotypes by targeting TRIM33 (21). A recent study by Li et al. reported that miR-629-5p could increase the invasiveness of tumor cells while increasing the permeability of endothelial cells, thereby promoting the invasion of lung adenocarcinoma (13). Zhu et al. found that miR-629 promotes the tumorigenesis of non-small-cell lung cancer by targeting FOXO1 and activating PI3K/AKT pathway (22). In addition, the study by Cheng et al. showed that the overexpression of miR-629-5p has immunosuppressive effect on the anti-tumor CD8^+^ T cells, which demonstrates its role in promoting cancer from a new perspective (23). However, it remains unclear whether miR-629-5p is involved in the regulation of PCa progression. In this study, we started with clinical data, and a series of functional experiments were preformed, whose results showed that miR-629-5p facilitated PCa cell malignant phenotype *in vitro* and vivo. Overall, these present results suggest that miR-629-5p works as an oncogene in PCa.

Downstream target genes, such as FOXO3, CXXC, PDCD4, SFRP2 and LRP6, which are directly affected by miR-629-5p, have been reported and verified by previous studies (11, 12, 24–26). In our study, however, a novel target gene of miR-629-5p was identified in PCa by dual-luciferase reporter assay. The A-Kinase Anchoring Protein (AKAPs) are a family of multivalent scaffolds that constrain signaling enzymes and effectors at subcellular locations to drive essential physiological events (27). Among them, AKAP4 and AKAP9 have been widely studied as cancer-promoting factors, while AKAP12 have proved to play the opposite role (28–30). AKAP13 serves as a scaffold protein for PKA and other transduction enzymes, and functions as a guanine nucleotide exchange factor for the small molecular weight GTPases RhoA and RhoC (31). Although it has been shown to be overexpressed in several cancers including esophageal cancers, breast cancer and hepatocellular carcinoma (32–34). According to a recent study, AKAP13 was regarded as a new tumor suppressor in PCa, which could prevent tumor invasion in collaboration with PTEN (35). However, its mechanism of action has not been deeply studied. In the present study, we confirmed this finding by knocking out AKAP13 in PCa cells and observed the phenotypic changes, and then verified its prognostic value in clinical data. More importantly, the silence of AKAP13 expression could reverse the tumor suppressor function of miR-629-5p inhibitor in PCa cells. Interestingly, we observed that its anti-cancer effect might be through promoting apoptosis.

To our knowledge, this study is the first research that provided the comprehensive evaluation of the role of miR-629-5p in PCa. There are still limitations in our current study that need to be taken into account. Firstly, we have verified the role of miR-629-5p in promoting tumor growth in animal models, but its role in promoting metastasis needs to be further confirmed. Secondly, some of the results in the public database do not support our conclusions, so it needs to be further verified by increasing the number of clinical samples. Finally, although it has been found that AKAP13 may be related to the apoptosis pathway, the specific mechanism of its function still needs further study.

In conclusion, our results demonstrated that high miR-629-5p expression is associated with the increase of malignant degree of PCa, and suggests a worse clinical prognosis. More precisely, the function of miR-629-5p tumor-promoting effect is realized by targeting AKAP13, which provides a new idea for clinical diagnosis and treatment of complex refractory PCa.

## Data Availability Statement

The original contributions presented in the study are included in the article/**Supplementary Material**. Further inquiries can be directed to the corresponding author.

## Ethics Statement

The studies involving human participants were reviewed and approved by the Ethics Committee of the First Affiliated Hospital for Guangzhou Medical University. The patients/participants provided their written informed consent to participate in this study. The animal study was reviewed and approved by the Animal Care and Use Committee of the First Affiliated Hospital of Guangzhou Medical University.

## Author Contributions

YL: project development, data analysis and manuscript writing. SZ and JW: experiment model establishment and data analysis. ZZhu and LL: cell culture and data collection. QX and MZ: histology examination and data collection. YM and ZW: data analysis. ZZhao: project development and manuscript editing. All authors contributed to the article and approved the submitted version.

## Funding

This work was supported by the grants from National Natural Science Foundation of China (No. 81572537 for ZZhao).

## Conflict of Interest

The authors declare that the research was conducted in the absence of any commercial or financial relationships that could be construed as a potential conflict of interest.

## Publisher’s Note

All claims expressed in this article are solely those of the authors and do not necessarily represent those of their affiliated organizations, or those of the publisher, the editors and the reviewers. Any product that may be evaluated in this article, or claim that may be made by its manufacturer, is not guaranteed or endorsed by the publisher.
