# Supplementary material for: MiR-629-5p Promotes Prostate Cancer Development and Metastasis by Targeting AKAP13
Source: Front Oncol. 2021 Oct 15;11:754353. doi: 10.3389/fonc.2021.754353 (PMC8554144; doi:10.3389/fonc.2021.754353)
Supplement: Supplementary file 1 [file DataSheet_1.zip › Supplementary Table 1.DOCX]

**Table S1.** Basic information of included datasets

| ID | Subset | Data type | country | Sample type | n (ANT/N) | n (PT) | n (MT) | n (T) |
| --- | --- | --- | --- | --- | --- | --- | --- | --- |
| GSE21032 | GSE21036 | miRNA | USA | Human tissues | 28 | 99 | 14 | 113 |
|  | GSE21034 | mRNA | USA | Human tissues | 29 | 131 | 19 | 150 |
| TCGA | - | miRNA | USA | Human tissues | 52 | 451 | 3 | 491 |
|  | - | mRNA | USA | Human tissues | 52 | 454 | 3 | 496 |

Abbreviations: ID, identification; n, number; ANT, adjacent normal tissues; N, normal tissues; PT, primary localized PCa tissues; MT, metastatic PCa tissues; T, tumor tissues.
