# Supplementary material for: MiR-629-5p Promotes Prostate Cancer Development and Metastasis by Targeting AKAP13
Source: Front Oncol. 2021 Oct 15;11:754353. doi: 10.3389/fonc.2021.754353 (PMC8554144; doi:10.3389/fonc.2021.754353)
Supplement: Supplementary file 1 [file DataSheet_1.zip › Supplementary Table 2.DOCX]

Table S2. Primer information used in the study

| Primer | Sequence（5’ to 3’） |
| --- | --- |
| AKAP13 Homo Forward | GTCAACGGGCACACTTTCAG |
| AKAP13 Homo Reverse | GGAGGCTAGACTTTCTCGGC |
| HEG1 Homo Forward | CAGTGGAATCGAGAAGAAACAGT |
| HEG1 Homo Reverse | ACGCAAAGACGTAAGTCCAAA |
| GADPH Homo Forward | ACAACTTTGGTATCGTGGAAGG |
| GADPH Homo Reverse | GCCATCACGCCACAGTTTC |
