# Supplementary material for: MiR-629-5p Promotes Prostate Cancer Development and Metastasis by Targeting AKAP13
Source: Front Oncol. 2021 Oct 15;11:754353. doi: 10.3389/fonc.2021.754353 (PMC8554144; doi:10.3389/fonc.2021.754353)
Supplement: Supplementary file 1 [file DataSheet_1.zip › Supplementary Table 3.DOCX]

**Table S3.** Differentially expressed miRNAs detected in clinical samples.

| miRNA_ID | Localized  PCa | Metastatic  PCa | up/down | log2  (fold change) | P-value |
| --- | --- | --- | --- | --- | --- |
| hsa-miR-204-3p | 264.7258 | 0 | down | -14.6922 | 6.98E-15 |
| hsa-miR-194-5p | 179.7608 | 0 | down | -14.1338 | 2.86E-13 |
| hsa-miR-144-5p | 66.7081 | 0 | down | -12.7036 | 2.48E-09 |
| hsa-miR-511-5p | 44.238 | 0 | down | -12.1111 | 7.60E-08 |
| hsa-miR-30c-1-3p | 31.5986 | 0 | down | -11.6256 | 1.03E-06 |
| hsa-miR-4326 | 30.1942 | 0 | down | -11.5601 | 1.72E-06 |
| hsa-miR-610 | 28.7898 | 0 | down | -11.4913 | 2.45E-06 |
| hsa-miR-4433b-5p | 28.0876 | 0 | down | -11.4557 | 2.94E-06 |
| hsa-miR-3620-5p | 26.6832 | 0 | down | -11.3817 | 4.28E-06 |
| hsa-miR-642a-3p | 26.6832 | 0 | down | -11.3817 | 4.28E-06 |
| hsa-miR-374a-5p | 25.981 | 0 | down | -11.3432 | 5.19E-06 |
| hsa-miR-1304-3p | 24.5767 | 0 | down | -11.2631 | 7.72E-06 |
| hsa-miR-3157-3p | 13.3416 | 0 | down | -10.3817 | 0.000391 |
| hsa-miR-5096 | 12.6394 | 0 | down | -10.3037 | 0.00053 |
| hsa-miR-942-5p | 11.9372 | 0 | down | -10.2212 | 0.000727 |
| hsa-miR-1255b-5p | 11.235 | 0 | down | -10.1338 | 0.001006 |
| hsa-miR-766-5p | 11.235 | 0 | down | -10.1338 | 0.001006 |
| hsa-miR-625-3p | 10.5329 | 0 | down | -10.0407 | 0.001409 |
| hsa-miR-26a-2-3p | 10.5329 | 0 | down | -10.0407 | 0.001409 |
| hsa-miR-15b-3p | 10.5329 | 0 | down | -10.0407 | 0.001409 |
| hsa-miR-4448 | 10.5329 | 0 | down | -10.0407 | 0.001409 |
| hsa-miR-18a-3p | 10.5329 | 0 | down | -10.0407 | 0.001409 |
| hsa-miR-20b-5p | 10.5329 | 0 | down | -10.0407 | 0.001409 |
| hsa-miR-372-3p | 10.5329 | 0 | down | -10.0407 | 0.001409 |
| hsa-miR-5189-5p | 9.8307 | 0 | down | -9.9412 | 0.001996 |
| hsa-miR-6852-3p | 9.8307 | 0 | down | -9.9412 | 0.001996 |
| hsa-miR-191-3p | 9.1285 | 0 | down | -9.8342 | 0.002864 |
| hsa-miR-548e-3p | 9.1285 | 0 | down | -9.8342 | 0.002864 |
| hsa-miR-324-5p | 9.1285 | 0 | down | -9.8342 | 0.002864 |
| hsa-miR-33a-5p | 9.1285 | 0 | down | -9.8342 | 0.002864 |
| hsa-miR-6813-5p | 8.4263 | 0 | down | -9.7188 | 0.004166 |
| hsa-miR-497-5p | 8.4263 | 0 | down | -9.7188 | 0.004166 |
| hsa-miR-1248 | 8.4263 | 0 | down | -9.7188 | 0.004166 |
| hsa-miR-4654 | 8.4263 | 0 | down | -9.7188 | 0.004166 |
| hsa-miR-2355-3p | 8.4263 | 0 | down | -9.7188 | 0.004166 |
| hsa-miR-1304-5p | 7.7241 | 0 | down | -9.5932 | 0.006149 |
| hsa-miR-1303 | 7.7241 | 0 | down | -9.5932 | 0.006149 |
| hsa-miR-5010-5p | 7.7241 | 0 | down | -9.5932 | 0.006149 |
| hsa-miR-6807-5p | 7.0219 | 0 | down | -9.4557 | 0.009224 |
| hsa-miR-421 | 7.0219 | 0 | down | -9.4557 | 0.009224 |
| hsa-miR-6810-5p | 7.0219 | 0 | down | -9.4557 | 0.009224 |
| hsa-let-7i-3p | 7.0219 | 0 | down | -9.4557 | 0.009224 |
| hsa-miR-323b-3p | 7.0219 | 0 | down | -9.4557 | 0.009224 |
| hsa-miR-3911 | 7.0219 | 0 | down | -9.4557 | 0.009224 |
| hsa-miR-664b-5p | 7.0219 | 0 | down | -9.4557 | 0.009224 |
| hsa-miR-769-3p | 7.0219 | 0 | down | -9.4557 | 0.009224 |
| hsa-miR-181c-3p | 7.0219 | 0 | down | -9.4557 | 0.009224 |
| hsa-miR-128-1-5p | 6.3197 | 0 | down | -9.3037 | 0.014079 |
| hsa-miR-339-5p | 6.3197 | 0 | down | -9.3037 | 0.014079 |
| hsa-miR-4498 | 6.3197 | 0 | down | -9.3037 | 0.014079 |
| hsa-miR-1227-3p | 6.3197 | 0 | down | -9.3037 | 0.014079 |
| hsa-miR-6727-5p | 6.3197 | 0 | down | -9.3037 | 0.014079 |
| hsa-miR-4661-5p | 6.3197 | 0 | down | -9.3037 | 0.014079 |
| hsa-miR-4745-5p | 6.3197 | 0 | down | -9.3037 | 0.014079 |
| hsa-miR-6770-3p | 6.3197 | 0 | down | -9.3037 | 0.014079 |
| hsa-miR-579-5p | 5.6175 | 0 | down | -9.1338 | 0.0219 |
| hsa-miR-6724-5p | 5.6175 | 0 | down | -9.1338 | 0.0219 |
| hsa-miR-6819-3p | 5.6175 | 0 | down | -9.1338 | 0.0219 |
| hsa-miR-6781-5p | 5.6175 | 0 | down | -9.1338 | 0.0219 |
| hsa-miR-29c-3p | 5.6175 | 0 | down | -9.1338 | 0.0219 |
| hsa-miR-4747-5p | 5.6175 | 0 | down | -9.1338 | 0.0219 |
| hsa-miR-30d-3p | 4.9153 | 0 | down | -8.9411 | 0.034783 |
| hsa-miR-3934-5p | 4.9153 | 0 | down | -8.9411 | 0.034783 |
| hsa-miR-193b-3p | 4.9153 | 0 | down | -8.9411 | 0.034783 |
| hsa-miR-361-5p | 4.9153 | 0 | down | -8.9411 | 0.034783 |
| hsa-miR-323a-5p | 4.9153 | 0 | down | -8.9411 | 0.034783 |
| hsa-miR-3173-5p | 4.9153 | 0 | down | -8.9411 | 0.034783 |
| hsa-miR-1976 | 4.9153 | 0 | down | -8.9411 | 0.034783 |
| hsa-miR-675-5p | 4.9153 | 0 | down | -8.9411 | 0.034783 |
| hsa-miR-7114-5p | 4.9153 | 0 | down | -8.9411 | 0.034783 |
| hsa-miR-4647 | 4.9153 | 0 | down | -8.9411 | 0.034783 |
| hsa-miR-181b-3p | 4.9153 | 0 | down | -8.9411 | 0.034783 |
| hsa-miR-6738-3p | 4.9153 | 0 | down | -8.9411 | 0.034783 |
| hsa-miR-4659a-3p | 4.9153 | 0 | down | -8.9411 | 0.034783 |
| hsa-miR-431-5p | 4.9153 | 0 | down | -8.9411 | 0.034783 |
| hsa-miR-3918 | 4.2131 | 0 | down | -8.7187 | 0.056522 |
| hsa-miR-3605-5p | 4.2131 | 0 | down | -8.7187 | 0.056522 |
| hsa-miR-6785-5p | 4.2131 | 0 | down | -8.7187 | 0.056522 |
| hsa-miR-598-3p | 4.2131 | 0 | down | -8.7187 | 0.056522 |
| hsa-miR-6805-5p | 4.2131 | 0 | down | -8.7187 | 0.056522 |
| hsa-miR-216b-5p | 4.2131 | 0 | down | -8.7187 | 0.056522 |
| hsa-miR-4791 | 4.2131 | 0 | down | -8.7187 | 0.056522 |
| hsa-miR-5589-5p | 4.2131 | 0 | down | -8.7187 | 0.056522 |
| hsa-miR-93-3p | 4.2131 | 0 | down | -8.7187 | 0.056522 |
| hsa-miR-6820-5p | 4.2131 | 0 | down | -8.7187 | 0.056522 |
| hsa-miR-4463 | 4.2131 | 0 | down | -8.7187 | 0.056522 |
| hsa-miR-3120-3p | 4.2131 | 0 | down | -8.7187 | 0.056522 |
| hsa-miR-5192 | 4.2131 | 0 | down | -8.7187 | 0.056522 |
| hsa-miR-377-5p | 4.2131 | 0 | down | -8.7187 | 0.056522 |
| hsa-miR-4672 | 4.2131 | 0 | down | -8.7187 | 0.056522 |
| hsa-miR-202-3p | 4.2131 | 0 | down | -8.7187 | 0.056522 |
| hsa-miR-520c-5p | 3.511 | 0 | down | -8.4557 | 0.094203 |
| hsa-miR-518d-5p | 3.511 | 0 | down | -8.4557 | 0.094203 |
| hsa-miR-4454 | 3.511 | 0 | down | -8.4557 | 0.094203 |
| hsa-miR-6817-3p | 3.511 | 0 | down | -8.4557 | 0.094203 |
| hsa-miR-4659a-5p | 3.511 | 0 | down | -8.4557 | 0.094203 |
| hsa-miR-487b-5p | 3.511 | 0 | down | -8.4557 | 0.094203 |
| hsa-miR-518f-5p | 3.511 | 0 | down | -8.4557 | 0.094203 |
| hsa-miR-4714-5p | 3.511 | 0 | down | -8.4557 | 0.094203 |
| hsa-miR-3120-5p | 3.511 | 0 | down | -8.4557 | 0.094203 |
| hsa-miR-3182 | 3.511 | 0 | down | -8.4557 | 0.094203 |
| hsa-miR-3179 | 3.511 | 0 | down | -8.4557 | 0.094203 |
| hsa-miR-802 | 3.511 | 0 | down | -8.4557 | 0.094203 |
| hsa-miR-18a-5p | 3.511 | 0 | down | -8.4557 | 0.094203 |
| hsa-miR-6826-5p | 3.511 | 0 | down | -8.4557 | 0.094203 |
| hsa-miR-6856-5p | 3.511 | 0 | down | -8.4557 | 0.094203 |
| hsa-miR-6750-5p | 3.511 | 0 | down | -8.4557 | 0.094203 |
| hsa-miR-3688-3p | 3.511 | 0 | down | -8.4557 | 0.094203 |
| hsa-miR-6500-3p | 3.511 | 0 | down | -8.4557 | 0.094203 |
| hsa-miR-526a | 3.511 | 0 | down | -8.4557 | 0.094203 |
| hsa-miR-32-3p | 3.511 | 0 | down | -8.4557 | 0.094203 |
| hsa-miR-4482-5p | 2.8088 | 0 | down | -8.1338 | 0.161491 |
| hsa-miR-148b-5p | 2.8088 | 0 | down | -8.1338 | 0.161491 |
| hsa-miR-4467 | 2.8088 | 0 | down | -8.1338 | 0.161491 |
| hsa-miR-6130 | 2.8088 | 0 | down | -8.1338 | 0.161491 |
| hsa-miR-5699-5p | 2.8088 | 0 | down | -8.1338 | 0.161491 |
| hsa-miR-6842-5p | 2.8088 | 0 | down | -8.1338 | 0.161491 |
| hsa-miR-505-3p | 2.8088 | 0 | down | -8.1338 | 0.161491 |
| hsa-miR-5088-5p | 2.8088 | 0 | down | -8.1338 | 0.161491 |
| hsa-miR-365b-5p | 2.8088 | 0 | down | -8.1338 | 0.161491 |
| hsa-miR-5684 | 2.8088 | 0 | down | -8.1338 | 0.161491 |
| hsa-miR-628-5p | 2.8088 | 0 | down | -8.1338 | 0.161491 |
| hsa-miR-7847-3p | 2.8088 | 0 | down | -8.1338 | 0.161491 |
| hsa-miR-6715b-3p | 2.8088 | 0 | down | -8.1338 | 0.161491 |
| hsa-miR-4658 | 2.8088 | 0 | down | -8.1338 | 0.161491 |
| hsa-miR-4645-3p | 2.8088 | 0 | down | -8.1338 | 0.161491 |
| hsa-miR-5010-3p | 2.8088 | 0 | down | -8.1338 | 0.161491 |
| hsa-miR-4748 | 2.8088 | 0 | down | -8.1338 | 0.161491 |
| hsa-miR-183-3p | 2.8088 | 0 | down | -8.1338 | 0.161491 |
| hsa-miR-629-3p | 2.8088 | 0 | down | -8.1338 | 0.161491 |
| hsa-miR-4676-3p | 2.8088 | 0 | down | -8.1338 | 0.161491 |
| hsa-miR-758-3p | 2.8088 | 0 | down | -8.1338 | 0.161491 |
| hsa-miR-1914-3p | 2.8088 | 0 | down | -8.1338 | 0.161491 |
| hsa-miR-5001-5p | 2.8088 | 0 | down | -8.1338 | 0.161491 |
| hsa-miR-4741 | 2.8088 | 0 | down | -8.1338 | 0.161491 |
| hsa-miR-4659b-3p | 2.8088 | 0 | down | -8.1338 | 0.161491 |
| hsa-miR-3131 | 2.8088 | 0 | down | -8.1338 | 0.161491 |
| hsa-miR-4685-3p | 2.8088 | 0 | down | -8.1338 | 0.161491 |
| hsa-miR-548ar-3p | 2.1066 | 0 | down | -7.7188 | 0.285714 |
| hsa-miR-548av-3p | 2.1066 | 0 | down | -7.7188 | 0.285714 |
| hsa-miR-548an | 2.1066 | 0 | down | -7.7188 | 0.285714 |
| hsa-miR-181c-5p | 2.1066 | 0 | down | -7.7188 | 0.285714 |
| hsa-miR-92a-1-5p | 2.1066 | 0 | down | -7.7188 | 0.285714 |
| hsa-miR-29b-1-5p | 2.1066 | 0 | down | -7.7188 | 0.285714 |
| hsa-miR-2278 | 2.1066 | 0 | down | -7.7188 | 0.285714 |
| hsa-miR-6847-5p | 2.1066 | 0 | down | -7.7188 | 0.285714 |
| hsa-miR-1275 | 2.1066 | 0 | down | -7.7188 | 0.285714 |
| hsa-miR-532-3p | 2.1066 | 0 | down | -7.7188 | 0.285714 |
| hsa-miR-6715a-3p | 2.1066 | 0 | down | -7.7188 | 0.285714 |
| hsa-miR-676-3p | 2.1066 | 0 | down | -7.7188 | 0.285714 |
| hsa-miR-6879-5p | 2.1066 | 0 | down | -7.7188 | 0.285714 |
| hsa-miR-151a-5p | 2.1066 | 0 | down | -7.7188 | 0.285714 |
| hsa-miR-3135a | 2.1066 | 0 | down | -7.7188 | 0.285714 |
| hsa-miR-141-3p | 2.1066 | 0 | down | -7.7188 | 0.285714 |
| hsa-miR-101-5p | 2.1066 | 0 | down | -7.7188 | 0.285714 |
| hsa-miR-376c-3p | 2.1066 | 0 | down | -7.7188 | 0.285714 |
| hsa-miR-628-3p | 2.1066 | 0 | down | -7.7188 | 0.285714 |
| hsa-miR-186-3p | 2.1066 | 0 | down | -7.7188 | 0.285714 |
| hsa-miR-516b-5p | 2.1066 | 0 | down | -7.7188 | 0.285714 |
| hsa-miR-487a-5p | 2.1066 | 0 | down | -7.7188 | 0.285714 |
| hsa-miR-4484 | 2.1066 | 0 | down | -7.7188 | 0.285714 |
| hsa-miR-4680-3p | 2.1066 | 0 | down | -7.7188 | 0.285714 |
| hsa-miR-4710 | 2.1066 | 0 | down | -7.7188 | 0.285714 |
| hsa-miR-6808-3p | 2.1066 | 0 | down | -7.7188 | 0.285714 |
| hsa-miR-363-5p | 2.1066 | 0 | down | -7.7188 | 0.285714 |
| hsa-miR-1296-5p | 2.1066 | 0 | down | -7.7188 | 0.285714 |
| hsa-miR-4523 | 2.1066 | 0 | down | -7.7188 | 0.285714 |
| hsa-miR-937-3p | 2.1066 | 0 | down | -7.7188 | 0.285714 |
| hsa-miR-223-5p | 995.7061 | 6.9852 | down | -7.1553 | 4.41E-14 |
| hsa-miR-548k | 1.4044 | 0 | down | -7.1338 | 0.52381 |
| hsa-miR-3620-3p | 1.4044 | 0 | down | -7.1338 | 0.52381 |
| hsa-miR-1236-5p | 1.4044 | 0 | down | -7.1338 | 0.52381 |
| hsa-miR-6736-5p | 1.4044 | 0 | down | -7.1338 | 0.52381 |
| hsa-miR-4648 | 1.4044 | 0 | down | -7.1338 | 0.52381 |
| hsa-miR-618 | 1.4044 | 0 | down | -7.1338 | 0.52381 |
| hsa-miR-6791-5p | 1.4044 | 0 | down | -7.1338 | 0.52381 |
| hsa-miR-4474-3p | 1.4044 | 0 | down | -7.1338 | 0.52381 |
| hsa-miR-3151-5p | 1.4044 | 0 | down | -7.1338 | 0.52381 |
| hsa-miR-6837-5p | 1.4044 | 0 | down | -7.1338 | 0.52381 |
| hsa-miR-4632-5p | 1.4044 | 0 | down | -7.1338 | 0.52381 |
| hsa-miR-561-5p | 1.4044 | 0 | down | -7.1338 | 0.52381 |
| hsa-miR-15a-5p | 1.4044 | 0 | down | -7.1338 | 0.52381 |
| hsa-miR-1273g-3p | 1.4044 | 0 | down | -7.1338 | 0.52381 |
| hsa-miR-4524a-3p | 1.4044 | 0 | down | -7.1338 | 0.52381 |
| hsa-miR-6511b-5p | 1.4044 | 0 | down | -7.1338 | 0.52381 |
| hsa-miR-942-3p | 1.4044 | 0 | down | -7.1338 | 0.52381 |
| hsa-miR-4660 | 1.4044 | 0 | down | -7.1338 | 0.52381 |
| hsa-miR-1910-3p | 1.4044 | 0 | down | -7.1338 | 0.52381 |
| hsa-miR-5001-3p | 1.4044 | 0 | down | -7.1338 | 0.52381 |
| hsa-miR-4510 | 1.4044 | 0 | down | -7.1338 | 0.52381 |
| hsa-miR-616-3p | 1.4044 | 0 | down | -7.1338 | 0.52381 |
| hsa-miR-6505-3p | 1.4044 | 0 | down | -7.1338 | 0.52381 |
| hsa-miR-5698 | 1.4044 | 0 | down | -7.1338 | 0.52381 |
| hsa-miR-29b-2-5p | 1.4044 | 0 | down | -7.1338 | 0.52381 |
| hsa-miR-132-3p | 1.4044 | 0 | down | -7.1338 | 0.52381 |
| hsa-miR-454-3p | 1.4044 | 0 | down | -7.1338 | 0.52381 |
| hsa-miR-6855-3p | 1.4044 | 0 | down | -7.1338 | 0.52381 |
| hsa-miR-146a-3p | 1.4044 | 0 | down | -7.1338 | 0.52381 |
| hsa-miR-2277-5p | 1.4044 | 0 | down | -7.1338 | 0.52381 |
| hsa-miR-1587 | 1.4044 | 0 | down | -7.1338 | 0.52381 |
| hsa-miR-16-2-3p | 1115.781 | 12.5734 | down | -6.4715 | 1.36E-12 |
| hsa-miR-16-5p | 345.4777 | 4.1911 | down | -6.3651 | 3.60E-11 |
| hsa-let-7d-3p | 190.9958 | 2.3284 | down | -6.3581 | 3.55E-10 |
| hsa-miR-340-5p | 150.971 | 1.8627 | down | -6.3407 | 1.06E-09 |
| hsa-miR-223-3p | 36.5139 | 0.4657 | down | -6.2929 | 2.85E-06 |
| hsa-miR-548j-5p | 0.7022 | 0 | down | -6.1338 | 1 |
| hsa-miR-340-3p | 0.7022 | 0 | down | -6.1338 | 1 |
| hsa-miR-5187-5p | 0.7022 | 0 | down | -6.1338 | 1 |
| hsa-miR-1827 | 0.7022 | 0 | down | -6.1338 | 1 |
| hsa-miR-519b-5p | 0.7022 | 0 | down | -6.1338 | 1 |
| hsa-miR-3944-5p | 0.7022 | 0 | down | -6.1338 | 1 |
| hsa-miR-3141 | 0.7022 | 0 | down | -6.1338 | 1 |
| hsa-miR-4749-5p | 0.7022 | 0 | down | -6.1338 | 1 |
| hsa-miR-523-5p | 0.7022 | 0 | down | -6.1338 | 1 |
| hsa-miR-6840-3p | 0.7022 | 0 | down | -6.1338 | 1 |
| hsa-miR-511-3p | 0.7022 | 0 | down | -6.1338 | 1 |
| hsa-miR-4721 | 0.7022 | 0 | down | -6.1338 | 1 |
| hsa-miR-1273g-5p | 0.7022 | 0 | down | -6.1338 | 1 |
| hsa-miR-539-5p | 0.7022 | 0 | down | -6.1338 | 1 |
| hsa-miR-1343-3p | 0.7022 | 0 | down | -6.1338 | 1 |
| hsa-miR-548u | 0.7022 | 0 | down | -6.1338 | 1 |
| hsa-miR-3150a-3p | 0.7022 | 0 | down | -6.1338 | 1 |
| hsa-miR-483-3p | 0.7022 | 0 | down | -6.1338 | 1 |
| hsa-miR-522-5p | 0.7022 | 0 | down | -6.1338 | 1 |
| hsa-miR-132-5p | 0.7022 | 0 | down | -6.1338 | 1 |
| hsa-miR-6772-3p | 0.7022 | 0 | down | -6.1338 | 1 |
| hsa-miR-590-3p | 0.7022 | 0 | down | -6.1338 | 1 |
| hsa-miR-518e-5p | 0.7022 | 0 | down | -6.1338 | 1 |
| hsa-miR-4788 | 0.7022 | 0 | down | -6.1338 | 1 |
| hsa-miR-548g-3p | 0.7022 | 0 | down | -6.1338 | 1 |
| hsa-miR-3127-3p | 0.7022 | 0 | down | -6.1338 | 1 |
| hsa-miR-4507 | 0.7022 | 0 | down | -6.1338 | 1 |
| hsa-miR-3168 | 0.7022 | 0 | down | -6.1338 | 1 |
| hsa-miR-3174 | 0.7022 | 0 | down | -6.1338 | 1 |
| hsa-miR-200b-5p | 0.7022 | 0 | down | -6.1338 | 1 |
| hsa-miR-214-5p | 0.7022 | 0 | down | -6.1338 | 1 |
| hsa-miR-6854-5p | 0.7022 | 0 | down | -6.1338 | 1 |
| hsa-miR-6726-5p | 0.7022 | 0 | down | -6.1338 | 1 |
| hsa-miR-200a-5p | 0.7022 | 0 | down | -6.1338 | 1 |
| hsa-miR-519c-5p | 0.7022 | 0 | down | -6.1338 | 1 |
| hsa-miR-135a-5p | 0.7022 | 0 | down | -6.1338 | 1 |
| hsa-miR-519a-5p | 0.7022 | 0 | down | -6.1338 | 1 |
| hsa-miR-3613-5p | 0.7022 | 0 | down | -6.1338 | 1 |
| hsa-miR-374c-5p | 0.7022 | 0 | down | -6.1338 | 1 |
| hsa-miR-187-3p | 0.7022 | 0 | down | -6.1338 | 1 |
| hsa-miR-34b-3p | 0.7022 | 0 | down | -6.1338 | 1 |
| hsa-miR-18b-5p | 0.7022 | 0 | down | -6.1338 | 1 |
| hsa-miR-512-3p | 0.7022 | 0 | down | -6.1338 | 1 |
| hsa-miR-4435 | 0.7022 | 0 | down | -6.1338 | 1 |
| hsa-miR-23b-5p | 21.0657 | 0.4657 | down | -5.4994 | 0.000157 |
| hsa-miR-485-5p | 58.984 | 1.397 | down | -5.3999 | 1.15E-06 |
| hsa-miR-215-5p | 108.8395 | 2.7941 | down | -5.2837 | 1.29E-07 |
| hsa-miR-32-5p | 16.1504 | 0.4657 | down | -5.116 | 0.000797 |
| hsa-let-7d-5p | 649.5262 | 20.4899 | down | -4.9864 | 9.60E-09 |
| hsa-miR-330-5p | 14.746 | 0.4657 | down | -4.9848 | 0.001334 |
| hsa-miR-6131 | 25.981 | 0.9314 | down | -4.8019 | 0.00016 |
| hsa-miR-7641 | 51.2599 | 1.8627 | down | -4.7824 | 9.85E-06 |
| hsa-miR-660-5p | 68.8147 | 2.7941 | down | -4.6223 | 5.75E-06 |
| hsa-miR-331-3p | 11.235 | 0.4657 | down | -4.5925 | 0.005479 |
| hsa-miR-543 | 21.7679 | 0.9314 | down | -4.5467 | 0.000497 |
| hsa-miR-4750-5p | 41.4292 | 1.8627 | down | -4.4752 | 4.68E-05 |
| hsa-miR-25-3p | 4069.194 | 186.2721 | down | -4.4493 | 1.21E-07 |
| hsa-miR-128-3p | 494.3421 | 22.8183 | down | -4.4372 | 2.63E-07 |
| hsa-miR-10b-5p | 2780.674 | 130.8562 | down | -4.4094 | 1.60E-07 |
| hsa-miR-499a-5p | 19.6613 | 0.9314 | down | -4.3998 | 0.000921 |
| hsa-miR-98-5p | 117.2658 | 5.5882 | down | -4.3913 | 3.10E-06 |
| hsa-let-7a-5p | 3361.386 | 160.6597 | down | -4.387 | 1.82E-07 |
| hsa-miR-342-3p | 16.8526 | 0.9314 | down | -4.1774 | 0.002228 |
| hsa-miR-1271-5p | 8.4263 | 0.4657 | down | -4.1774 | 0.019787 |
| hsa-miR-92a-3p | 1550.437 | 86.6165 | down | -4.1619 | 7.93E-07 |
| hsa-miR-224-5p | 31.5986 | 1.8627 | down | -4.0844 | 0.000295 |
| hsa-miR-4732-5p | 7.7241 | 0.4657 | down | -4.0519 | 0.027969 |
| hsa-miR-20a-5p | 88.476 | 6.0538 | down | -3.8694 | 4.37E-05 |
| hsa-miR-30e-3p | 120.7768 | 8.3822 | down | -3.8489 | 2.87E-05 |
| hsa-miR-484 | 111.6483 | 7.9166 | down | -3.8179 | 3.68E-05 |
| hsa-miR-126-3p | 4333.92 | 308.2804 | down | -3.8134 | 5.11E-06 |
| hsa-miR-192-5p | 3449.16 | 253.3301 | down | -3.7672 | 6.70E-06 |
| hsa-miR-197-3p | 12.6394 | 0.9314 | down | -3.7624 | 0.009775 |
| hsa-miR-139-5p | 270.3433 | 20.0243 | down | -3.755 | 1.48E-05 |
| hsa-let-7a-3p | 43.5358 | 3.2598 | down | -3.7393 | 0.00032 |
| hsa-miR-140-5p | 30.1942 | 2.3284 | down | -3.6969 | 0.000871 |
| hsa-miR-455-3p | 5.6175 | 0.4657 | down | -3.5925 | 0.084472 |
| hsa-miR-4487 | 5.6175 | 0.4657 | down | -3.5925 | 0.084472 |
| hsa-miR-6515-5p | 139.0337 | 11.642 | down | -3.578 | 7.82E-05 |
| hsa-miR-26a-5p | 2908.473 | 246.3449 | down | -3.5615 | 2.12E-05 |
| hsa-miR-659-5p | 31.5986 | 2.7941 | down | -3.4994 | 0.001306 |
| hsa-miR-200a-3p | 40.0249 | 3.7254 | down | -3.4254 | 0.000985 |
| hsa-miR-148b-3p | 1167.041 | 110.3662 | down | -3.4025 | 5.55E-05 |
| hsa-miR-4306 | 4.9153 | 0.4657 | down | -3.3998 | 0.12496 |
| hsa-miR-450a-5p | 23.8745 | 2.3284 | down | -3.3581 | 0.004026 |
| hsa-miR-199b-5p | 23.8745 | 2.3284 | down | -3.3581 | 0.004026 |
| hsa-miR-574-3p | 46.3446 | 4.6568 | down | -3.315 | 0.001061 |
| hsa-miR-374a-3p | 18.257 | 1.8627 | down | -3.293 | 0.008475 |
| hsa-miR-19b-3p | 170.6323 | 17.6959 | down | -3.2694 | 0.000211 |
| hsa-miR-27a-3p | 685.3379 | 71.7148 | down | -3.2565 | 0.00013 |
| hsa-miR-148a-3p | 29443.55 | 3215.522 | down | -3.1948 | 0.000143 |
| hsa-miR-34a-5p | 4.2131 | 0.4657 | down | -3.1774 | 0.186957 |
| hsa-let-7g-5p | 2590.381 | 295.2413 | down | -3.1332 | 0.000207 |
| hsa-miR-10a-5p | 1193.724 | 136.91 | down | -3.1242 | 0.000229 |
| hsa-miR-182-5p | 110.2439 | 13.5047 | down | -3.0292 | 0.000992 |
| hsa-let-7f-5p | 3160.559 | 392.1028 | down | -3.0109 | 0.000379 |
| hsa-miR-4440 | 11.235 | 1.397 | down | -3.0076 | 0.038199 |
| hsa-miR-26b-5p | 268.2368 | 33.529 | down | -3 | 0.000594 |
| hsa-miR-23a-5p | 131.3096 | 17.2302 | down | -2.93 | 0.001317 |
| hsa-miR-335-3p | 7.0219 | 0.9314 | down | -2.9144 | 0.099976 |
| hsa-miR-136-3p | 3.511 | 0.4657 | down | -2.9144 | 0.282609 |
| hsa-miR-126-5p | 94.0935 | 12.5734 | down | -2.9037 | 0.001841 |
| hsa-miR-155-5p | 37.2161 | 5.1225 | down | -2.861 | 0.005989 |
| hsa-miR-101-3p | 1150.89 | 162.0567 | down | -2.8282 | 0.000987 |
| hsa-miR-10b-3p | 9.8307 | 1.397 | down | -2.815 | 0.06403 |
| hsa-miR-200c-3p | 9.8307 | 1.397 | down | -2.815 | 0.06403 |
| hsa-miR-122-5p | 136415.2 | 20131.36 | down | -2.7605 | 0.001236 |
| hsa-miR-199b-3p | 497.8531 | 74.5088 | down | -2.7402 | 0.00164 |
| hsa-miR-199a-3p | 497.8531 | 74.5088 | down | -2.7402 | 0.00164 |
| hsa-miR-30e-5p | 373.5653 | 59.6071 | down | -2.6478 | 0.00255 |
| hsa-miR-196b-5p | 8.4263 | 1.397 | down | -2.5926 | 0.109379 |
| hsa-miR-30c-2-3p | 8.4263 | 1.397 | down | -2.5926 | 0.109379 |
| hsa-miR-147b | 25.2789 | 4.1911 | down | -2.5925 | 0.023091 |
| hsa-miR-6780a-5p | 5.6175 | 0.9314 | down | -2.5925 | 0.191276 |
| hsa-miR-654-3p | 5.6175 | 0.9314 | down | -2.5925 | 0.191276 |
| hsa-miR-1260b | 2.8088 | 0.4657 | down | -2.5925 | 0.430642 |
| hsa-let-7b-5p | 21194.22 | 3629.512 | down | -2.5458 | 0.003316 |
| hsa-miR-584-5p | 129.203 | 22.3527 | down | -2.5311 | 0.005768 |
| hsa-miR-29a-3p | 97.6045 | 17.2302 | down | -2.502 | 0.008165 |
| hsa-miR-27b-3p | 1108.057 | 200.7082 | down | -2.4649 | 0.005051 |
| hsa-miR-451a | 22352.83 | 4179.481 | down | -2.4191 | 0.005759 |
| hsa-miR-21-5p | 3970.185 | 750.211 | down | -2.4038 | 0.006216 |
| hsa-miR-3130-3p | 157.9929 | 30.7349 | down | -2.3619 | 0.0103 |
| hsa-miR-142-5p | 139.0337 | 27.4751 | down | -2.3392 | 0.011815 |
| hsa-miR-183-5p | 135.5228 | 27.4751 | down | -2.3023 | 0.013625 |
| hsa-miR-6741-5p | 70.9212 | 14.4361 | down | -2.2965 | 0.020344 |
| hsa-miR-296-3p | 36.5139 | 7.4509 | down | -2.293 | 0.031275 |
| hsa-miR-206 | 56.1752 | 11.642 | down | -2.2706 | 0.025072 |
| hsa-miR-199a-5p | 506.9815 | 109.9005 | down | -2.2057 | 0.015245 |
| hsa-miR-548o-3p | 23.1723 | 5.1225 | down | -2.1775 | 0.067852 |
| hsa-miR-3074-5p | 6.3197 | 1.397 | down | -2.1775 | 0.251568 |
| hsa-miR-450b-5p | 4.2131 | 0.9314 | down | -2.1774 | 0.372947 |
| hsa-miR-10a-3p | 2.1066 | 0.4657 | down | -2.1774 | 0.658385 |
| hsa-miR-363-3p | 348.9887 | 79.6313 | down | -2.1318 | 0.021118 |
| hsa-miR-493-5p | 11.9372 | 2.7941 | down | -2.095 | 0.14853 |
| hsa-let-7e-5p | 75.1344 | 17.6959 | down | -2.0861 | 0.03439 |
| hsa-miR-30d-5p | 2962.542 | 703.1772 | down | -2.0749 | 0.023213 |
| hsa-miR-1301-3p | 43.5358 | 10.7106 | down | -2.0232 | 0.058972 |
| hsa-miR-221-3p | 1050.477 | 260.781 | down | -2.0101 | 0.030224 |
| hsa-miR-181a-5p | 322.3054 | 81.4941 | down | -1.9837 | 0.036005 |
| hsa-miR-100-5p | 1185.298 | 305.4863 | down | -1.9561 | 0.036615 |
| hsa-miR-96-5p | 7.0219 | 1.8627 | down | -1.9145 | 0.305221 |
| hsa-miR-23a-3p | 251.3842 | 67.058 | down | -1.9064 | 0.047325 |
| hsa-miR-424-3p | 48.4511 | 13.039 | down | -1.8937 | 0.079888 |
| hsa-miR-30a-3p | 62.495 | 17.2302 | down | -1.8588 | 0.08052 |
| hsa-miR-885-3p | 33.7051 | 9.3136 | down | -1.8556 | 0.102406 |
| hsa-miR-501-3p | 318.7945 | 88.9449 | down | -1.8416 | 0.058238 |
| hsa-let-7i-5p | 14001.68 | 4003.454 | down | -1.8063 | 0.059507 |
| hsa-miR-146b-5p | 294.2178 | 87.0822 | down | -1.7564 | 0.077217 |
| hsa-miR-326 | 89.8804 | 27.0095 | down | -1.7345 | 0.093913 |
| hsa-miR-4433b-3p | 591.2444 | 179.2869 | down | -1.7215 | 0.081308 |
| hsa-miR-374b-5p | 10.5329 | 3.2598 | down | -1.692 | 0.302645 |
| hsa-miR-22-5p | 133.4162 | 41.4455 | down | -1.6866 | 0.104558 |
| hsa-miR-486-3p | 464.8501 | 150.8804 | down | -1.6234 | 0.111381 |
| hsa-miR-142-3p | 83.5607 | 27.4751 | down | -1.6047 | 0.140065 |
| hsa-miR-1307-5p | 44.9402 | 14.9018 | down | -1.5925 | 0.174324 |
| hsa-miR-34c-5p | 16.8526 | 5.5882 | down | -1.5925 | 0.2718 |
| hsa-miR-3940-5p | 2.8088 | 0.9314 | down | -1.5925 | 0.726708 |
| hsa-miR-449a | 1.4044 | 0.4657 | down | -1.5925 | 1 |
| hsa-miR-4433a-3p | 1.4044 | 0.4657 | down | -1.5925 | 1 |
| hsa-miR-424-5p | 1.4044 | 0.4657 | down | -1.5925 | 1 |
| hsa-miR-505-5p | 1.4044 | 0.4657 | down | -1.5925 | 1 |
| hsa-miR-3960 | 47.749 | 16.2988 | down | -1.5507 | 0.189492 |
| hsa-miR-24-2-5p | 18.9591 | 6.5195 | down | -1.5401 | 0.282111 |
| hsa-miR-6729-5p | 16.1504 | 5.5882 | down | -1.5311 | 0.307713 |
| hsa-miR-30a-5p | 631.9714 | 218.8697 | down | -1.5298 | 0.143864 |
| hsa-miR-493-3p | 92.6891 | 32.5976 | down | -1.5076 | 0.173729 |
| hsa-miR-125b-1-3p | 34.4073 | 12.1077 | down | -1.5068 | 0.225552 |
| hsa-miR-125b-5p | 108.1373 | 38.1858 | down | -1.5018 | 0.175178 |
| hsa-miR-3199 | 35.1095 | 12.5734 | down | -1.4815 | 0.236841 |
| hsa-miR-381-3p | 71.6234 | 26.5438 | down | -1.4321 | 0.21955 |
| hsa-miR-24-3p | 1653.659 | 621.6832 | down | -1.4114 | 0.195286 |
| hsa-miR-409-3p | 428.3362 | 162.5224 | down | -1.3981 | 0.207658 |
| hsa-miR-30c-5p | 124.2877 | 47.9651 | down | -1.3736 | 0.240106 |
| hsa-miR-411-5p | 7.0219 | 2.7941 | down | -1.3295 | 0.580436 |
| hsa-miR-184 | 3.511 | 1.397 | down | -1.3295 | 0.769726 |
| hsa-miR-99b-5p | 493.6399 | 196.9828 | down | -1.3254 | 0.248203 |
| hsa-miR-144-3p | 95.4979 | 38.6515 | down | -1.3049 | 0.283342 |
| hsa-miR-532-5p | 1688.768 | 696.6577 | down | -1.2774 | 0.273721 |
| hsa-miR-342-5p | 77.9431 | 32.5976 | down | -1.2577 | 0.324783 |
| hsa-miR-1228-5p | 417.8033 | 178.8212 | down | -1.2243 | 0.314487 |
| hsa-miR-150-5p | 85.6672 | 37.2544 | down | -1.2013 | 0.355805 |
| hsa-miR-423-3p | 1103.843 | 481.5134 | down | -1.1969 | 0.331362 |
| hsa-miR-6501-5p | 33.003 | 14.4361 | down | -1.1929 | 0.420445 |
| hsa-miR-425-3p | 142.5447 | 62.4012 | down | -1.1918 | 0.348578 |
| hsa-miR-3615 | 2105.167 | 925.3067 | down | -1.1859 | 0.338328 |
| hsa-miR-1180-3p | 456.4238 | 201.6396 | down | -1.1786 | 0.35071 |
| hsa-miR-23b-3p | 75.8366 | 33.529 | down | -1.1775 | 0.384492 |
| hsa-miR-195-3p | 6.3197 | 2.7941 | down | -1.1775 | 0.700311 |
| hsa-miR-181a-3p | 6.3197 | 2.7941 | down | -1.1775 | 0.700311 |
| hsa-miR-203a-3p | 2.1066 | 0.9314 | down | -1.1774 | 1 |
| hsa-miR-323a-3p | 2.1066 | 0.9314 | down | -1.1774 | 1 |
| hsa-miR-320e | 160.0994 | 71.2491 | down | -1.168 | 0.368302 |
| hsa-miR-127-3p | 2636.725 | 1173.98 | down | -1.1673 | 0.352385 |
| hsa-miR-93-5p | 186.0805 | 82.8911 | down | -1.1666 | 0.370993 |
| hsa-miR-4429 | 293.5156 | 130.8562 | down | -1.1655 | 0.361906 |
| hsa-miR-486-5p | 41095 | 18553.17 | down | -1.1473 | 0.367209 |
| hsa-miR-181d-5p | 33.003 | 14.9018 | down | -1.1471 | 0.455728 |
| hsa-miR-320c | 11103.04 | 5017.705 | down | -1.1459 | 0.368572 |
| hsa-miR-145-3p | 27.3854 | 12.5734 | down | -1.123 | 0.516859 |
| hsa-miR-6845-5p | 7.0219 | 3.2598 | down | -1.1071 | 0.725047 |
| hsa-miR-125a-5p | 54.7709 | 25.6124 | down | -1.0966 | 0.454224 |
| hsa-miR-320b | 25653.83 | 12141.22 | down | -1.0793 | 0.424606 |
| hsa-miR-4669 | 114.457 | 54.4846 | down | -1.0709 | 0.462953 |
| hsa-miR-150-3p | 139.0337 | 66.5923 | down | -1.062 | 0.455737 |
| hsa-miR-30b-5p | 7.7241 | 3.7254 | down | -1.052 | 0.745855 |
| hsa-miR-378j | 7.7241 | 3.7254 | down | -1.052 | 0.745855 |
| hsa-miR-130b-3p | 23.8745 | 11.642 | down | -1.0361 | 0.603651 |
| hsa-miR-181a-2-3p | 56.1752 | 27.4751 | down | -1.0318 | 0.51106 |
| hsa-miR-222-3p | 178.3564 | 87.5479 | down | -1.0266 | 0.492216 |
| hsa-miR-186-5p | 885.4622 | 437.2738 | down | -1.0179 | 0.483858 |
| hsa-miR-939-5p | 101.8176 | 50.2935 | down | -1.0175 | 0.51148 |
| hsa-miR-6862-5p | 8.4263 | 17.2302 | up | 1.032 | 0.055867 |
| hsa-miR-2110 | 922.6783 | 1906.961 | up | 1.0474 | 0.016829 |
| hsa-miR-483-5p | 782.9424 | 1638.729 | up | 1.0656 | 0.015719 |
| hsa-miR-3150b-3p | 43.5358 | 92.2047 | up | 1.0826 | 0.020289 |
| hsa-miR-4489 | 7.0219 | 14.9018 | up | 1.0856 | 0.056561 |
| hsa-miR-130a-3p | 9.1285 | 19.5586 | up | 1.0994 | 0.047081 |
| hsa-miR-542-3p | 8.4263 | 18.1615 | up | 1.1079 | 0.044263 |
| hsa-miR-3064-5p | 5.6175 | 12.1077 | up | 1.1079 | 0.066846 |
| hsa-miR-362-5p | 6.3197 | 13.9704 | up | 1.1444 | 0.052986 |
| hsa-miR-27a-5p | 117.2658 | 261.2466 | up | 1.1556 | 0.012322 |
| hsa-miR-339-3p | 244.3623 | 546.243 | up | 1.1605 | 0.011247 |
| hsa-miR-1254 | 11.235 | 25.1467 | up | 1.1624 | 0.031275 |
| hsa-miR-185-5p | 26352.51 | 59391 | up | 1.1723 | 0.010081 |
| hsa-miR-146b-3p | 21.0657 | 47.4994 | up | 1.173 | 0.018361 |
| hsa-miR-3177-3p | 11.9372 | 27.4751 | up | 1.2027 | 0.025705 |
| hsa-miR-378d | 3553.084 | 8194.576 | up | 1.2056 | 0.008826 |
| hsa-miR-378i | 396.7376 | 922.5127 | up | 1.2174 | 0.008729 |
| hsa-miR-4497 | 640.3977 | 1492.04 | up | 1.2202 | 0.008471 |
| hsa-miR-378b | 28.0876 | 66.1266 | up | 1.2353 | 0.014416 |
| hsa-miR-422a | 716.9365 | 1708.581 | up | 1.2529 | 0.007376 |
| hsa-miR-3158-3p | 595.4575 | 1452.457 | up | 1.2864 | 0.006467 |
| hsa-miR-4800-5p | 4.9153 | 12.1077 | up | 1.3006 | 0.044792 |
| hsa-miR-378f | 613.7145 | 1513.927 | up | 1.3027 | 0.006021 |
| hsa-miR-210-3p | 345.4777 | 868.4937 | up | 1.3299 | 0.005462 |
| hsa-miR-378a-3p | 166737.2 | 423189.8 | up | 1.3437 | 0.004904 |
| hsa-miR-28-3p | 749.2372 | 1949.803 | up | 1.3798 | 0.004282 |
| hsa-miR-196a-5p | 0.7022 | 1.8627 | up | 1.4074 | 0.430642 |
| hsa-miR-2277-3p | 0.7022 | 1.8627 | up | 1.4074 | 0.430642 |
| hsa-miR-3198 | 9.8307 | 27.0095 | up | 1.4581 | 0.012434 |
| hsa-miR-140-3p | 3118.428 | 8568.983 | up | 1.4583 | 0.002977 |
| hsa-miR-378g | 748.5351 | 2090.905 | up | 1.482 | 0.002722 |
| hsa-miR-4734 | 15.4482 | 43.3083 | up | 1.4872 | 0.006872 |
| hsa-miR-3190-3p | 2.8088 | 7.9166 | up | 1.4949 | 0.057273 |
| hsa-miR-3187-5p | 3.511 | 10.245 | up | 1.545 | 0.034945 |
| hsa-miR-582-3p | 71.6234 | 209.5561 | up | 1.5488 | 0.002546 |
| hsa-miR-7854-3p | 1.4044 | 4.1911 | up | 1.5774 | 0.137874 |
| hsa-miR-185-3p | 457.126 | 1397.507 | up | 1.6122 | 0.00153 |
| hsa-miR-6128 | 2.1066 | 6.5195 | up | 1.6298 | 0.06403 |
| hsa-miR-490-3p | 9.8307 | 30.7349 | up | 1.6445 | 0.005997 |
| hsa-miR-933 | 2.8088 | 8.8479 | up | 1.6554 | 0.036521 |
| hsa-miR-766-3p | 3.511 | 11.1763 | up | 1.6705 | 0.023711 |
| hsa-miR-195-5p | 0.7022 | 2.3284 | up | 1.7294 | 0.282609 |
| hsa-miR-6850-5p | 16.8526 | 58.6757 | up | 1.7998 | 0.001739 |
| hsa-miR-378e | 463.4457 | 1628.018 | up | 1.8126 | 0.000585 |
| hsa-miR-378h | 74.4322 | 264.5064 | up | 1.8293 | 0.000676 |
| hsa-miR-9-5p | 1.4044 | 5.1225 | up | 1.8669 | 0.072973 |
| hsa-miR-873-3p | 58.2818 | 216.0757 | up | 1.8904 | 0.000555 |
| hsa-miR-636 | 2.1066 | 8.3822 | up | 1.9924 | 0.023239 |
| hsa-miR-31-5p | 0.7022 | 2.7941 | up | 1.9924 | 0.186957 |
| hsa-miR-3180 | 56.1752 | 235.1685 | up | 2.0657 | 0.000235 |
| hsa-miR-4665-5p | 87.0716 | 367.4218 | up | 2.0772 | 0.000198 |
| hsa-miR-6859-5p | 4.9153 | 21.4213 | up | 2.1237 | 0.00271 |
| hsa-miR-125b-2-3p | 43.5358 | 191.3946 | up | 2.1363 | 0.000189 |
| hsa-miR-27b-5p | 4.2131 | 19.0929 | up | 2.1801 | 0.002612 |
| hsa-miR-491-5p | 14.0438 | 63.7982 | up | 2.1836 | 0.000351 |
| hsa-miR-4738-3p | 19.6613 | 89.4106 | up | 2.1851 | 0.000233 |
| hsa-miR-4479 | 0.7022 | 3.2598 | up | 2.2148 | 0.12496 |
| hsa-miR-136-5p | 9.1285 | 43.3083 | up | 2.2462 | 0.000469 |
| hsa-miR-3196 | 17.5548 | 86.6165 | up | 2.3028 | 0.000145 |
| hsa-miR-5585-3p | 2.8088 | 15.8331 | up | 2.4949 | 0.001948 |
| hsa-miR-629-5p | 2452.049 | 14534.35 | up | 2.5674 | 1.03E-05 |
| hsa-miR-345-3p | 3.511 | 20.9556 | up | 2.5774 | 0.000871 |
| hsa-miR-3928-3p | 151.6731 | 963.4925 | up | 2.6673 | 7.04E-06 |
| hsa-miR-6799-5p | 2.8088 | 18.1615 | up | 2.6929 | 0.000851 |
| hsa-miR-7855-5p | 2.1066 | 14.4361 | up | 2.7767 | 0.001419 |
| hsa-miR-452-5p | 30.8964 | 263.1094 | up | 3.0902 | 1.30E-06 |
| hsa-miR-576-3p | 434.6559 | 3764.56 | up | 3.1145 | 4.50E-07 |
| hsa-miR-455-5p | 2.1066 | 18.6272 | up | 3.1444 | 0.000291 |
| hsa-miR-1287-5p | 17.5548 | 156.9343 | up | 3.1602 | 1.34E-06 |
| hsa-miR-2115-5p | 1.4044 | 12.5734 | up | 3.1623 | 0.001141 |
| hsa-miR-1306-3p | 7.0219 | 63.7982 | up | 3.1836 | 6.65E-06 |
| hsa-miR-6882-5p | 2.1066 | 19.5586 | up | 3.2148 | 0.000211 |
| hsa-miR-6839-5p | 1.4044 | 13.5047 | up | 3.2654 | 0.000747 |
| hsa-miR-3195 | 0.7022 | 6.9852 | up | 3.3143 | 0.007447 |
| hsa-miR-4488 | 387.6091 | 3874.926 | up | 3.3215 | 1.29E-07 |
| hsa-miR-4535 | 0.7022 | 7.4509 | up | 3.4075 | 0.005479 |
| hsa-miR-4532 | 39.3227 | 482.9105 | up | 3.6183 | 4.31E-08 |
| hsa-miR-4664-3p | 4.9153 | 67.9893 | up | 3.79 | 5.83E-07 |
| hsa-miR-1183 | 0.7022 | 10.7106 | up | 3.931 | 0.000797 |
| hsa-miR-4421 | 1.4044 | 37.2544 | up | 4.7294 | 5.82E-07 |
| hsa-miR-4492 | 28.0876 | 770.7009 | up | 4.7782 | 3.70E-11 |
| hsa-miR-1246 | 1644.53 | 76260.27 | up | 5.5352 | 6.57E-14 |
| hsa-miR-935 | 0 | 0.4657 | up | 5.5413 | 1 |
| hsa-miR-4271 | 0 | 0.4657 | up | 5.5413 | 1 |
| hsa-miR-6758-3p | 0 | 0.4657 | up | 5.5413 | 1 |
| hsa-miR-4430 | 0 | 0.4657 | up | 5.5413 | 1 |
| hsa-miR-222-5p | 0 | 0.4657 | up | 5.5413 | 1 |
| hsa-miR-548az-3p | 0 | 0.4657 | up | 5.5413 | 1 |
| hsa-miR-449c-5p | 0 | 0.4657 | up | 5.5413 | 1 |
| hsa-miR-202-5p | 0 | 0.4657 | up | 5.5413 | 1 |
| hsa-miR-6827-5p | 0 | 0.4657 | up | 5.5413 | 1 |
| hsa-miR-548n | 0 | 0.4657 | up | 5.5413 | 1 |
| hsa-let-7f-2-3p | 0 | 0.4657 | up | 5.5413 | 1 |
| hsa-miR-29b-3p | 0 | 0.4657 | up | 5.5413 | 1 |
| hsa-miR-409-5p | 0 | 0.4657 | up | 5.5413 | 1 |
| hsa-miR-9-3p | 0 | 0.4657 | up | 5.5413 | 1 |
| hsa-let-7f-1-3p | 0 | 0.4657 | up | 5.5413 | 1 |
| hsa-miR-369-5p | 0 | 0.4657 | up | 5.5413 | 1 |
| hsa-miR-365a-5p | 0 | 0.4657 | up | 5.5413 | 1 |
| hsa-miR-3129-3p | 0 | 0.4657 | up | 5.5413 | 1 |
| hsa-miR-5701 | 0 | 0.4657 | up | 5.5413 | 1 |
| hsa-miR-6505-5p | 0 | 0.4657 | up | 5.5413 | 1 |
| hsa-miR-329-3p | 0 | 0.4657 | up | 5.5413 | 1 |
| hsa-miR-539-3p | 0 | 0.4657 | up | 5.5413 | 1 |
| hsa-miR-4531 | 0 | 0.4657 | up | 5.5413 | 1 |
| hsa-miR-329-5p | 0 | 0.4657 | up | 5.5413 | 1 |
| hsa-miR-24-1-5p | 0 | 0.4657 | up | 5.5413 | 1 |
| hsa-miR-147a | 0 | 0.4657 | up | 5.5413 | 1 |
| hsa-miR-431-3p | 0 | 0.4657 | up | 5.5413 | 1 |
| hsa-miR-138-5p | 0 | 0.4657 | up | 5.5413 | 1 |
| hsa-miR-4483 | 0 | 0.4657 | up | 5.5413 | 1 |
| hsa-miR-301a-5p | 0 | 0.4657 | up | 5.5413 | 1 |
| hsa-miR-4293 | 0 | 0.4657 | up | 5.5413 | 1 |
| hsa-let-7b-3p | 0 | 0.9314 | up | 6.5413 | 0.52381 |
| hsa-miR-6779-5p | 0 | 0.9314 | up | 6.5413 | 0.52381 |
| hsa-miR-487b-3p | 0 | 0.9314 | up | 6.5413 | 0.52381 |
| hsa-miR-149-5p | 0 | 0.9314 | up | 6.5413 | 0.52381 |
| hsa-miR-16-1-3p | 0 | 0.9314 | up | 6.5413 | 0.52381 |
| hsa-miR-3681-5p | 0 | 0.9314 | up | 6.5413 | 0.52381 |
| hsa-miR-216a-3p | 0 | 0.9314 | up | 6.5413 | 0.52381 |
| hsa-miR-7974 | 0 | 0.9314 | up | 6.5413 | 0.52381 |
| hsa-miR-1290 | 1.4044 | 148.0863 | up | 6.7203 | 2.71E-12 |
| hsa-miR-889-3p | 0 | 1.397 | up | 7.1262 | 0.285714 |
| hsa-miR-3665 | 0 | 1.397 | up | 7.1262 | 0.285714 |
| hsa-miR-338-3p | 0 | 1.397 | up | 7.1262 | 0.285714 |
| hsa-miR-4657 | 0 | 1.397 | up | 7.1262 | 0.285714 |
| hsa-miR-204-5p | 0 | 1.397 | up | 7.1262 | 0.285714 |
| hsa-miR-365b-3p | 0 | 1.8627 | up | 7.5413 | 0.161491 |
| hsa-miR-365a-3p | 0 | 1.8627 | up | 7.5413 | 0.161491 |
| hsa-miR-3653-3p | 0 | 2.3284 | up | 7.8632 | 0.094203 |
| hsa-miR-382-3p | 0 | 2.3284 | up | 7.8632 | 0.094203 |
| hsa-miR-1269b | 0 | 2.7941 | up | 8.1262 | 0.056522 |
| hsa-miR-4526 | 0 | 2.7941 | up | 8.1262 | 0.056522 |
| hsa-miR-6796-5p | 0 | 3.2598 | up | 8.3486 | 0.034783 |
| hsa-miR-494-3p | 0 | 3.2598 | up | 8.3486 | 0.034783 |
| hsa-miR-4425 | 0 | 3.7254 | up | 8.5413 | 0.0219 |
| hsa-miR-217 | 0 | 4.1911 | up | 8.7112 | 0.014079 |
| hsa-miR-580-3p | 0 | 4.6568 | up | 8.8632 | 0.009224 |
| hsa-miR-3185 | 0 | 5.5882 | up | 9.1262 | 0.004166 |
| hsa-miR-4437 | 0 | 5.5882 | up | 9.1262 | 0.004166 |
| hsa-miR-6804-5p | 0 | 6.0538 | up | 9.2417 | 0.002864 |
| hsa-miR-5006-5p | 0 | 6.0538 | up | 9.2417 | 0.002864 |
| hsa-miR-1226-5p | 0 | 6.9852 | up | 9.4482 | 0.001409 |
| hsa-miR-632 | 0 | 7.9166 | up | 9.6287 | 0.000727 |
| hsa-miR-6761-5p | 0 | 7.9166 | up | 9.6287 | 0.000727 |
| hsa-miR-6775-3p | 0 | 8.3822 | up | 9.7112 | 0.00053 |
| hsa-miR-3127-5p | 0 | 9.3136 | up | 9.8632 | 0.000291 |
| hsa-miR-585-3p | 0 | 9.7793 | up | 9.9336 | 0.000218 |
| hsa-miR-4743-5p | 0 | 9.7793 | up | 9.9336 | 0.000218 |
| hsa-miR-615-5p | 0 | 9.7793 | up | 9.9336 | 0.000218 |
| hsa-miR-6891-5p | 0 | 10.245 | up | 10.0007 | 0.000165 |
| hsa-miR-3191-3p | 0 | 10.245 | up | 10.0007 | 0.000165 |
| hsa-miR-4682 | 0 | 13.039 | up | 10.3486 | 3.57E-05 |
| hsa-miR-1273e | 0 | 13.5047 | up | 10.3992 | 2.82E-05 |
| hsa-miR-6793-5p | 0 | 13.9704 | up | 10.4482 | 2.25E-05 |
| hsa-miR-6764-5p | 0 | 14.4361 | up | 10.4955 | 1.80E-05 |
| hsa-miR-378c | 0 | 16.2988 | up | 10.6706 | 7.72E-06 |
| hsa-miR-219a-1-3p | 0 | 17.2302 | up | 10.7507 | 5.19E-06 |
| hsa-miR-6514-5p | 0 | 18.6272 | up | 10.8632 | 2.94E-06 |
| hsa-miR-197-5p | 0 | 19.0929 | up | 10.8988 | 2.45E-06 |
| hsa-miR-26b-3p | 0 | 19.0929 | up | 10.8988 | 2.45E-06 |
| hsa-miR-6861-5p | 0 | 19.0929 | up | 10.8988 | 2.45E-06 |
| hsa-miR-6745 | 0 | 25.6124 | up | 11.3226 | 2.97E-07 |
| hsa-miR-4646-5p | 0 | 26.5438 | up | 11.3742 | 2.23E-07 |
| hsa-miR-1288-3p | 0 | 53.0876 | up | 12.3742 | 5.92E-10 |
