# Supplementary material for: MiR-629-5p Promotes Prostate Cancer Development and Metastasis by Targeting AKAP13
Source: Front Oncol. 2021 Oct 15;11:754353. doi: 10.3389/fonc.2021.754353 (PMC8554144; doi:10.3389/fonc.2021.754353)
Supplement: Supplementary file 1 [file DataSheet_1.zip › Supplementary Table 4.DOCX]

**Table S4.** Differentially expressed miRNAs in TCGA database identified by bioinformatics analysis.

| miRNAs | logFC | AveExpr | t | P.Value | adj.P.Val | B | regulated |
| --- | --- | --- | --- | --- | --- | --- | --- |
| hsa-miR-375 | 3.069777 | 16.18358 | 19.99477 | 4.44E-67 | 7.20E-64 | 141.9941 | Up-Regulated |
| hsa-miR-182-5p | 2.574049 | 13.74812 | 18.23515 | 2.53E-58 | 2.05E-55 | 121.9638 | Up-Regulated |
| hsa-miR-25-3p | 1.484203 | 12.41468 | 17.59293 | 3.58E-55 | 1.94E-52 | 114.7557 | Up-Regulated |
| hsa-miR-200c-3p | 2.034048 | 13.25981 | 17.44037 | 1.99E-54 | 6.46E-52 | 113.0534 | Up-Regulated |
| hsa-miR-183-5p | 2.415184 | 11.9856 | 17.17337 | 3.95E-53 | 1.07E-50 | 110.0842 | Up-Regulated |
| hsa-miR-148a-3p | 1.842789 | 15.60983 | 17.01133 | 2.41E-52 | 5.59E-50 | 108.2888 | Up-Regulated |
| hsa-miR-93-5p | 2.023915 | 11.42387 | 16.28587 | 7.38E-49 | 1.50E-46 | 100.3164 | Up-Regulated |
| hsa-miR-96-5p | 2.214455 | 4.43151 | 15.97774 | 2.15E-47 | 3.89E-45 | 96.96565 | Up-Regulated |
| hsa-miR-103a-3p | 1.212921 | 13.68271 | 15.41817 | 9.30E-45 | 1.51E-42 | 90.94038 | Up-Regulated |
| hsa-miR-423-5p | 1.668159 | 5.312865 | 14.98118 | 1.00E-42 | 1.48E-40 | 86.2933 | Up-Regulated |
| hsa-miR-92a-3p | 1.459785 | 12.6194 | 14.66417 | 2.89E-41 | 3.91E-39 | 82.95672 | Up-Regulated |
| hsa-miR-708-3p | 1.510494 | 4.063505 | 13.61359 | 1.59E-36 | 1.98E-34 | 72.12932 | Up-Regulated |
| hsa-miR-500a-3p | 1.486865 | 7.068173 | 13.54287 | 3.26E-36 | 3.78E-34 | 71.4142 | Up-Regulated |
| hsa-miR-17-5p | 1.738868 | 7.847826 | 13.47226 | 6.68E-36 | 7.23E-34 | 70.70196 | Up-Regulated |
| hsa-miR-425-5p | 1.710715 | 6.334499 | 13.23448 | 7.40E-35 | 7.50E-33 | 68.31744 | Up-Regulated |
| hsa-miR-146b-5p | 1.681611 | 7.651562 | 13.09468 | 3.01E-34 | 2.87E-32 | 66.9256 | Up-Regulated |
| hsa-miR-153-5p | 2.403398 | 4.415071 | 13.04019 | 5.19E-34 | 4.68E-32 | 66.38523 | Up-Regulated |
| hsa-miR-20a-5p | 1.844685 | 7.592857 | 12.99889 | 7.84E-34 | 6.70E-32 | 65.9764 | Up-Regulated |
| hsa-miR-342-3p | 1.233343 | 6.185711 | 12.92491 | 1.64E-33 | 1.33E-31 | 65.24572 | Up-Regulated |
| hsa-miR-191-5p | 1.281098 | 8.352926 | 12.65818 | 2.29E-32 | 1.77E-30 | 62.62995 | Up-Regulated |
| hsa-miR-141-5p | 1.246217 | 8.637258 | 12.45307 | 1.71E-31 | 1.26E-29 | 60.63865 | Up-Regulated |
| hsa-miR-141-3p | 1.780662 | 9.528524 | 11.8267 | 7.04E-29 | 4.97E-27 | 54.67177 | Up-Regulated |
| hsa-miR-7-1-3p | 1.310837 | 4.012577 | 11.74649 | 1.50E-28 | 1.02E-26 | 53.92067 | Up-Regulated |
| hsa-miR-1307-3p | 1.273435 | 8.106112 | 11.64248 | 4.00E-28 | 2.60E-26 | 52.95116 | Up-Regulated |
| hsa-miR-708-5p | 1.462288 | 2.965971 | 11.61383 | 5.29E-28 | 3.30E-26 | 52.67375 | Up-Regulated |
| hsa-miR-501-3p | 1.283815 | 3.841539 | 11.45277 | 2.41E-27 | 1.45E-25 | 51.1748 | Up-Regulated |
| hsa-miR-21-5p | 1.034686 | 16.58518 | 11.34657 | 6.30E-27 | 3.52E-25 | 50.22141 | Up-Regulated |
| hsa-miR-153-3p | 1.521825 | 2.364664 | 11.21769 | 2.17E-26 | 1.18E-24 | 49.01101 | Up-Regulated |
| hsa-miR-19b-3p | 1.56575 | 6.997627 | 11.18131 | 2.88E-26 | 1.51E-24 | 48.7157 | Up-Regulated |
| hsa-miR-20a-3p | 1.201753 | 2.085733 | 10.87845 | 4.52E-25 | 2.29E-23 | 45.99244 | Up-Regulated |
| hsa-miR-146b-3p | 1.203181 | 4.42054 | 10.61916 | 4.59E-24 | 2.19E-22 | 43.69911 | Up-Regulated |
| hsa-miR-629-5p | 1.08425 | 6.086999 | 10.52571 | 1.06E-23 | 4.90E-22 | 42.87356 | Up-Regulated |
| hsa-miR-106b-5p | 1.019921 | 7.342391 | 10.49603 | 1.36E-23 | 6.14E-22 | 42.62276 | Up-Regulated |
| hsa-miR-3074-5p | 1.338236 | 2.241969 | 10.48334 | 1.52E-23 | 6.68E-22 | 42.51234 | Up-Regulated |
| hsa-miR-5586-5p | 1.099462 | 1.720653 | 10.42996 | 2.64E-23 | 1.13E-21 | 42.01053 | Up-Regulated |
| hsa-miR-106a-5p | 1.709925 | 6.147134 | 10.39427 | 3.33E-23 | 1.38E-21 | 41.73948 | Up-Regulated |
| hsa-miR-17-3p | 1.002131 | 7.90126 | 10.36239 | 4.39E-23 | 1.78E-21 | 41.464 | Up-Regulated |
| hsa-miR-126-3p | 1.235911 | 10.12415 | 10.06742 | 5.64E-22 | 2.23E-20 | 38.94172 | Up-Regulated |
| hsa-miR-30b-5p | 1.158757 | 8.954479 | 9.586711 | 3.24E-20 | 1.03E-18 | 34.93927 | Up-Regulated |
| hsa-miR-93-3p | 1.047366 | 2.464444 | 9.471139 | 8.41E-20 | 2.53E-18 | 33.99761 | Up-Regulated |
| hsa-miR-660-5p | 1.106556 | 5.592776 | 9.276262 | 4.12E-19 | 1.20E-17 | 32.42838 | Up-Regulated |
| hsa-miR-199b-5p | 1.16254 | 7.104345 | 8.861833 | 1.12E-17 | 2.76E-16 | 29.17073 | Up-Regulated |
| hsa-miR-200c-5p | 1.085084 | 4.006162 | 8.63873 | 6.34E-17 | 1.49E-15 | 27.46303 | Up-Regulated |
| hsa-miR-20b-5p | 1.558713 | 7.143456 | 8.594944 | 8.88E-17 | 2.03E-15 | 27.13173 | Up-Regulated |
| hsa-miR-362-5p | 1.019011 | 2.731262 | 8.075138 | 4.37E-15 | 9.08E-14 | 23.29805 | Up-Regulated |
| hsa-miR-3065-3p | 1.182394 | 5.329113 | 8.0396 | 5.66E-15 | 1.16E-13 | 23.04275 | Up-Regulated |
| hsa-miR-19a-3p | 1.184996 | 4.006166 | 7.753343 | 4.44E-14 | 8.18E-13 | 21.01891 | Up-Regulated |
| hsa-miR-3607-3p | 1.059372 | 4.396035 | 5.26394 | 2.03E-07 | 1.90E-06 | 6.050738 | Up-Regulated |
| hsa-miR-9-5p | 1.140705 | 9.062029 | 5.113016 | 4.40E-07 | 3.82E-06 | 5.305903 | Up-Regulated |
| hsa-miR-210-3p | 1.16293 | 5.709498 | 4.730829 | 2.85E-06 | 2.20E-05 | 3.508226 | Up-Regulated |
| hsa-miR-23c | -1.91831 | 1.213315 | -17.9818 | 6.05E-55 | 2.46E-52 | 114.2741 | Down-Regulated |
| hsa-miR-143-3p | -1.04031 | 18.30043 | -9.95072 | 1.53E-21 | 5.76E-20 | 37.95755 | Down-Regulated |
| hsa-miR-187-3p | -2.05064 | 2.538594 | -9.81018 | 5.38E-21 | 1.94E-19 | 36.71468 | Down-Regulated |
| hsa-miR-133b | -1.53162 | 4.032255 | -9.61896 | 2.53E-20 | 8.54E-19 | 35.18639 | Down-Regulated |
| hsa-miR-221-3p | -1.23541 | 7.41663 | -9.56912 | 3.75E-20 | 1.17E-18 | 34.79539 | Down-Regulated |
| hsa-miR-184 | -1.40877 | 1.360241 | -8.18215 | 2.84E-15 | 5.98E-14 | 23.75904 | Down-Regulated |
| hsa-miR-136-3p | -1.04379 | 3.429746 | -7.39328 | 5.45E-13 | 9.02E-12 | 18.55712 | Down-Regulated |
| hsa-miR-490-3p | -1.19686 | 1.297401 | -5.965 | 7.84E-09 | 8.78E-08 | 9.373563 | Down-Regulated |
| hsa-miR-888-5p | -1.0182 | 0.597068 | -4.40909 | 3.34E-05 | 0.000219 | 1.847435 | Down-Regulated |
| hsa-miR-891b | -1.34309 | 0.650179 | -4.30223 | 9.69E-05 | 0.000596 | 1.111483 | Down-Regulated |
| hsa-miR-205-5p | -1.19166 | 8.810716 | -3.8037 | 0.000159 | 0.00094 | -0.31213 | Down-Regulated |
| hsa-miR-892b | -1.01957 | 0.580026 | -3.75642 | 0.000635 | 0.003369 | -0.57658 | Down-Regulated |
| hsa-miR-892c-3p | -1.15454 | 0.678972 | -3.45384 | 0.00164 | 0.007992 | -1.43763 | Down-Regulated |

Abbreviations: FC, fold change.
