# Supplementary material for: MiR-629-5p Promotes Prostate Cancer Development and Metastasis by Targeting AKAP13
Source: Front Oncol. 2021 Oct 15;11:754353. doi: 10.3389/fonc.2021.754353 (PMC8554144; doi:10.3389/fonc.2021.754353)
Supplement: Supplementary file 1 [file DataSheet_1.zip › Supplementary Table 5.DOCX]

**Table S5.** Identifying the expression of hsa-miR-629-5p in other tumors by dbDEMC 3.0

| **ID** | **Cancer Type** | **Cancer Subtype** | **Design** | **logFC** | **Status** |
| --- | --- | --- | --- | --- | --- |
| TCGA_BRCA | breast cancer | breast invasive carcinoma | cancer vs normal | 0.10 | UP |
| TCGA_KIRP | kidney cancer | kidney renal papillary cell carcinoma | cancer vs normal | 1.09 | UP |
| TCGA_KIRP | kidney cancer | kidney renal papillary cell carcinoma | high grade vs low grade | 0.59 | UP |
| TCGA_KIRC | kidney cancer | kidney renal clear cell carcinoma | cancer vs normal | 1.60 | UP |
| TCGA_KICH | kidney cancer | kidney chromophobe cancer | cancer vs normal | 0.69 | UP |
| TCGA_LUAD | lung cancer | lung adenocarcinoma | cancer vs normal | 0.74 | UP |
| TCGA_LUSC | lung cancer | lung squamous cell carcinoma | cancer vs normal | 0.72 | UP |
| TCGA_UCEC | endometrial cancer | uterine corpus endometrial carcinoma | cancer vs normal | 0.43 | UP |
| TCGA_THCA | thyroid cancer | thyroid carcinoma | cancer vs normal | 0.49 | UP |
| TCGA_PRAD | prostate cancer | prostate adenocarcinoma | cancer vs normal | 0.69 | UP |
| TCGA_PAAD | pancreatic cancer | pancreatic adenocarcinoma | high grade vs low grade | 0.56 | UP |
| TCGA_ESCA | esophageal cancer | esophageal carcinoma | cancer vs normal | 0.62 | UP |
| TCGA_SKCM | melanoma | skin cutaneous melanoma | metastasis | 0.38 | UP |
| TCGA_BLCA | bladder cancer | bladder urothelial carcinoma | cancer vs normal | 0.86 | UP |

Abbreviations: ID, identification; FC, fold change.
